# Replicative senescence and high glucose induce the accrual of self-derived cytosolic nucleic acids in human endothelial cells

**DOI:** 10.1038/s41420-024-01954-z

**Published:** 2024-04-20

**Authors:** Deborah Ramini, Angelica Giuliani, Katarzyna Malgorzata Kwiatkowska, Michele Guescini, Gianluca Storci, Emanuela Mensà, Rina Recchioni, Luciano Xumerle, Elisa Zago, Jacopo Sabbatinelli, Spartaco Santi, Paolo Garagnani, Massimiliano Bonafè, Fabiola Olivieri

**Affiliations:** 1 Clinic of Laboratory and Precision Medicine, IRCCS INRCA, Ancona, Italy; 2 Department of Clinical and Molecular Sciences, DISCLIMO, Università Politecnica delle Marche, Ancona, Italy; 3 Department of Medical and Surgical Sciences (DIMEC), University of Bologna, Bologna, Italy; 4 Department of Biomolecular Science, University of Urbino Carlo Bo, Urbino, Italy; 5 IRCCS Azienda Ospedaliero Universitaria di Bologna, Bologna, Italy; 6 Personal Genomics s.r.l, Verona, Italy; 7 CNR Institute of Molecular Genetics “Luigi Luca Cavalli-Sforza” - Unit of Bologna, Bologna, Italy; 8 IRCCS Istituto Ortopedico Rizzoli, Bologna, Italy

**Keywords:** Senescence, Mechanisms of disease

## Abstract

Recent literature shows that loss of replicative ability and acquisition of a proinflammatory secretory phenotype in senescent cells is coupled with the build-in of nucleic acids in the cytoplasm. Its implication in human age-related diseases is under scrutiny. In human endothelial cells (ECs), we assessed the accumulation of intracellular nucleic acids during in vitro replicative senescence and after exposure to high glucose concentrations, which mimic an in vivo condition of hyperglycemia. We showed that exposure to high glucose induces senescent-like features in ECs, including telomere shortening and proinflammatory cytokine release, coupled with the accrual in the cytoplasm of telomeres, double-stranded DNA and RNA (dsDNA, dsRNA), as well as RNA:DNA hybrid molecules. Senescent ECs showed an activation of the dsRNA sensors RIG-I and MDA5 and of the DNA sensor TLR9, which was not paralleled by the involvement of the canonical (cGAS) and non-canonical (IFI16) activation of the STING pathway. Under high glucose conditions, only a sustained activation of TLR9 was observed. Notably, senescent cells exhibit increased proinflammatory cytokine (IL-1β, IL-6, IL-8) production without a detectable secretion of type I interferon (IFN), a phenomenon that can be explained, at least in part, by the accumulation of methyl-adenosine containing RNAs. At variance, exposure to exogenous nucleic acids enhances both IL-6 and IFN-β1 expression in senescent cells. This study highlights the accrual of cytoplasmic nucleic acids as a marker of senescence-related endothelial dysfunction, that may play a role in dysmetabolic age-related diseases.

## Introduction

The main features of senescent cells are the loss of replicative ability and the acquisition of a secretory phenotype (SASP), characterized by the release of numerous cytokines, chemokines, and growth factors [1]. An increased release of nuclear and mitochondrial DNA fragments into the cytoplasm has been described in senescent cells, in association with the activation of the cytosolic DNA sensors, cyclic GMP-AMP Synthase (cGAS) and toll-like receptor (TLR)-9 [2, 3]. These sensors can activate downstream pathways to release inflammatory cytokines and activate type I interferon (IFN-I) responses [4]. While the presence of RNA in cytoplasm is not unexpected, the build-in of cytoplasmic self-derived double-stranded RNAs (RNAs) was recently shown [5]. Similarly to what occurs to the cytoplasm accrual of DNA fragments, the activation of innate immune pathways ensues [6]. Most of the studies on the accumulation of nucleic acids are aimed at understanding the mechanisms of autoimmune disease or cancer [7, 8]. Nevertheless, activation of inflammation and type I IFN pathways has been associated with inflammaging, the low-grade systemic inflammation that acts as the main driver of age-related dysfunction and that is a central player in a variety of age-related diseases (ARDs) [9–11]. However, no data on the proinflammatory effects of misplaced nucleic acids in cellular models of age-related endothelial dysfunction, such as that observed in type 2 diabetes (T2D), were provided to date. Human endothelial cellular models are useful to disentangle the pathophysiological role of dysfunctional endothelium in the development of cardiovascular (CV) disease and organ damage in T2D [12]. We previously reported that high glucose treatment is associated with the induction of a senescent phenotype [13] and the accrual of cytoplasmic dsRNA [14] in human umbilical vein endothelial cells (HUVECs). In this regard, we previously hypothesized that an unbalanced response to misplaced nucleic acid in endothelial cells, could foster inflammaging, and promote the development and progression of the most common ARDs [9, 11, 15]. Based on this evidence, we aimed to better investigate the combined effects of replicative senescence and high glucose on the accumulation of endogenous cytoplasmic nucleic acids, their possible loading onto extracellular vesicles (EVs), and how the presence of such self-derived cytosolic nucleic acids affects the ability of endothelial cells to respond to exogenous nucleic acids.

## Results

### Characterization of HUVEC senescence

**Fig. 1:**
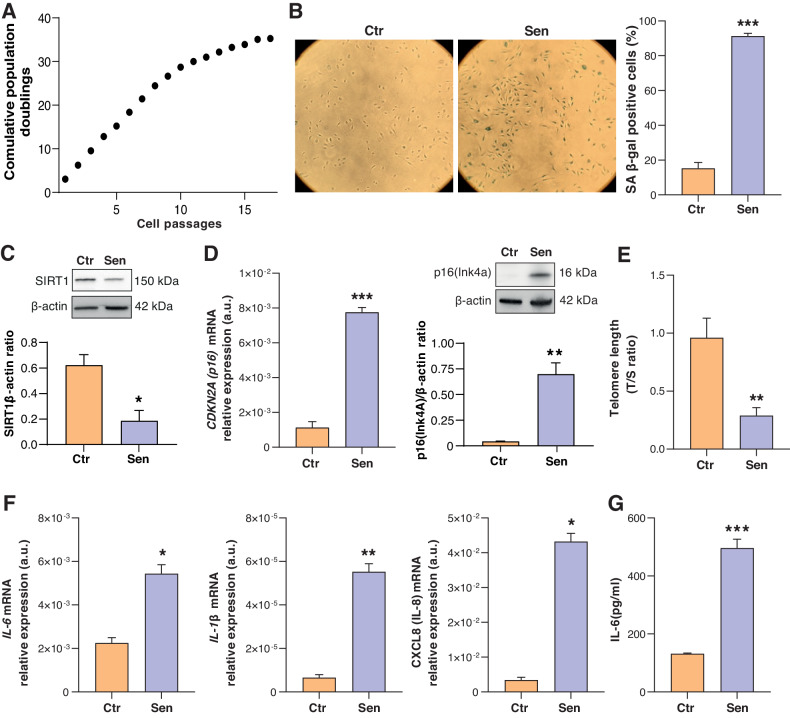
Characterization of replicative senescence in human umbilical vein endothelial cells (HUVECs). **A** Growth curve showing cumulative population doublings (CPDs, Y axes) of HUVECs undergoing replicative Senescence (cell passages, X axes). **B** Representative images and quantification of the Senescence-Associated β-Galactosidase (SA β-Gal) staining positivity in young (Ctr, SA β-Gal < 10%) and Senescent (Sen, SA β-Gal > 80%) HUVECs. **C** Western blot and densitometric analysis of SIRT1 in Ctr and Sen cells. Protein expression values are reported as SIRT1/β-actin ratio. **D** p16(INK4a) mRNA relative expression in arbitrary units (a.u.) in Ctr and Sen cells obtained through Real Time PCR. Data were normalized using β-actin as internal control. Western blot and densitometric analysis of p16(Ink4a) in Ctr and Sen cells. Protein expression values are reported as p16(Ink4)/β-actin ratio. **E** Telomere length was analyzed by Real Time-PCR calculated as telomere/single copy gene ratio (T/S). **F** IL-6, IL-8, and IL-1 β mRNA relative expression in arbitrary units (a.u.) in CTR and SEN cells obtained through Real Time PCR. Data were normalized using β-actin as internal control. **G** IL-6 concentration (pg/ml) in the culture medium of Ctr and Sen cells. Data are mean ± SD of three independent experiments. *t test *p* < 0.05; **t test *p* < 0.01; ***t test *p* < 0.001.

Compared to low passage control (CPD = 15, Ctr) cells, extensively passaged senescent (CPD = 35, Sen) cells revealed growth arrest (Fig. 1A), increased SA β-gal activity (Fig. 1B), decreased sirtuin-1 (SIRT1) expression (Fig. 1C), upregulation of the cell cycle regulator p16(CDKN2A) both at the transcriptional and translational levels (Fig. 1D), progressive telomere shortening (Fig. 1E), upregulation of mRNAs coding for the pro-inflammatory interleukins IL‐6, IL-1β and IL‐8 (Fig. 1F), and a significantly higher release of IL-6 in conditioned medium (Fig. 1G).

### High glucose promotes the acquisition of senescence features in endothelial cells

**Fig. 2:**
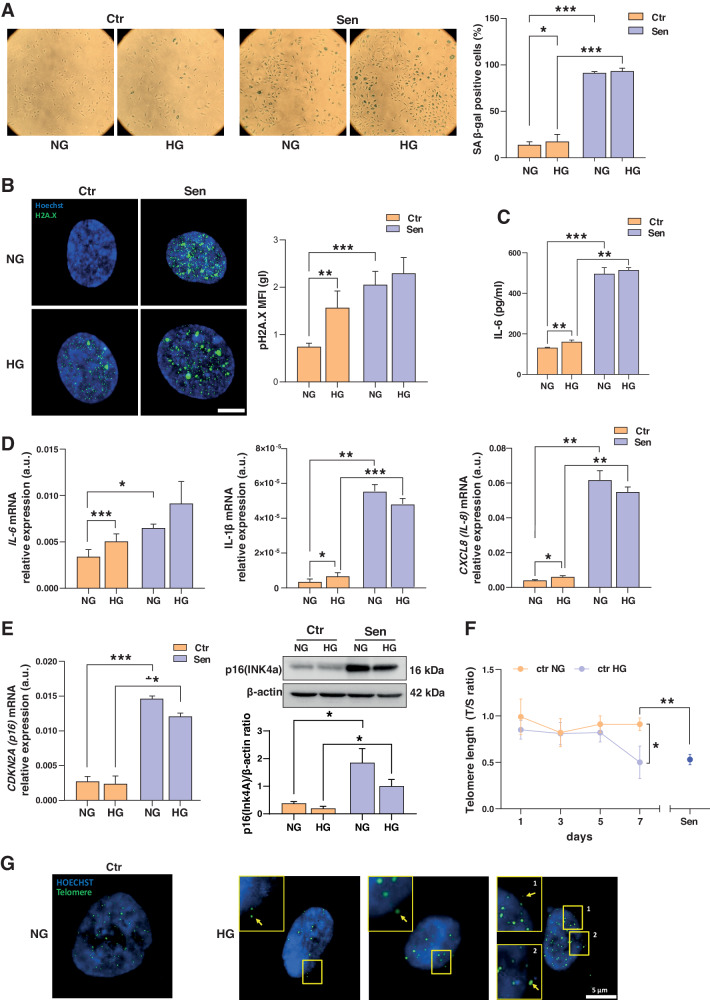
High glucose promotes senescence feature acquisition in HUVECs. **A** Representative images and quantification of the Senescence-Associated β-Galactosidase (SA β-Gal) staining positivity in Ctr and Sen HUVECs in presence of normal glucose (NG) or high glucose (HG) concentration in culture medium. **B** Representative immunofluorescence (IF) images and quantification of Ctr and Sen HUVECs in NG and HG conditions labeled with an antibody against h2ax-phosphorylated (green fluorescence) and Hoechst for nuclei staining (blue fluorescence). **C** IL-6 concentration (pg/ml) in the culture medium in Ctr and Sen cells. **D** IL-6, IL-8, and IL-1 β mRNA relative expression in arbitrary units (a.u.) in Ctr and Sen cells obtained through Real Time PCR. Data were normalized using β-actin as internal control. **E** p16(INK4a) mRNA relative expression in arbitrary units (a.u.) in Ctr and Sen cells in NG and HG conditions obtained through Real Time PCR. Data were normalized using β-actin as internal control. Western blot and densitometric analysis of p16(Ink4a) in Ctr and Sen cells. Protein expression values are reported as p16(Ink4)/β-actin ratio. **F** Telomere length was analyzed by Real Time-PCR calculated as telomere/single copy gene ratio (T/S) at day 1, 3, 5, and 7 of young cells in NG or HG condition. Telomere length was compared to Sen cells. **G** Representative IF images of telomeric FISH probes (green fluorescence) and Hoechst for nuclei staining (blue fluorescence). Yellow arrows indicate telomeric probes in cell cytoplasm. Data are mean ± SD of three independent experiments. *t test *p* < 0.05; **t test *p* < 0.01; ***t test *p* < 0.001.

Exposure of Ctr cells to HG for 7 days elicited increased levels of several senescence biomarkers, namely SA β-gal activity, pH2A.X histone phosphorylation, IL-6 mRNA and protein, and IL-8 and IL-1β mRNAs (Fig. 2A–D). No significant modulation of p16(CDKN2A) mRNA and protein was observed under high glucose conditions (Fig. 2E). Telomere length was significantly decreased after 7 days of culture in HG medium, with Ctr HG cells showing a telomere shortening comparable to that of Sen cells (Fig. 2F). Notably, telomere DNA detection by FISH showed the presence of intranuclear telomere sequences, but also cytosolic signals in HG-treated Ctr cells (Fig. 2G). These results suggest that exposure to HG induces a senescent-like phenotype in HUVEC cells, including the replication-independent trimming of telomere ends.

### Transcriptome analysis of Ctr and Sen cells

**Fig. 3:**
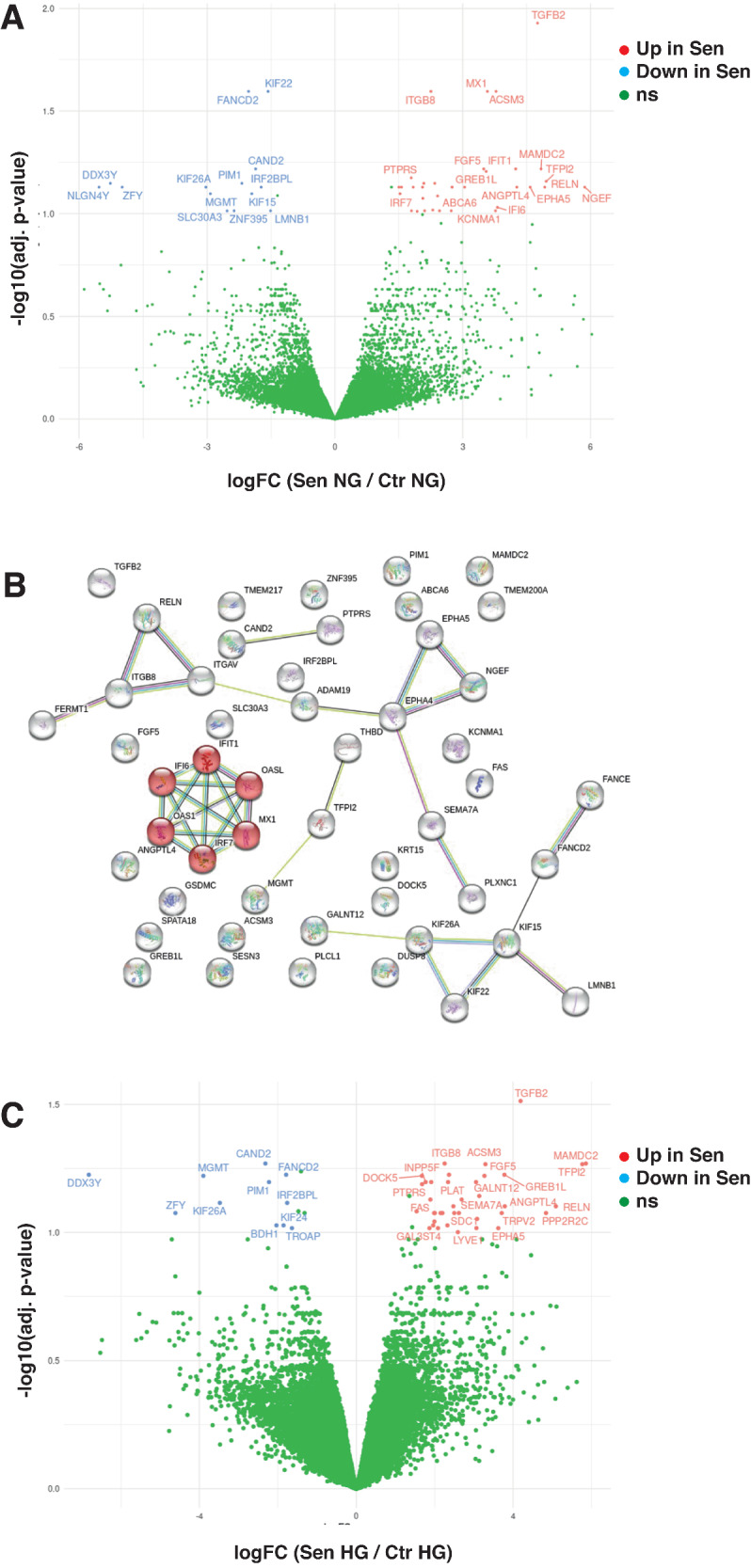
RNA-sequencing analysis of HUVECs. **A** Volcano plot depicting the impact of Senescence on the gene expression patterns in normal glucose levels. Some of the DE gene names were omitted due to the overlapping labels. In blue: downregulated DE genes; in red: upregulated DE genes; in green: non-significant (ns) genes. **B** Results of functional protein network analysis of 48 genes differentially expressed (adj. p-value < 0.10 and logFC > 1.5) in Senescent cells compared to young in normal glucose culture media. In red are highlighted proteins-players involved in interferon Alpha/Beta signaling. Nodes correspond to transcripts and edges represent protein–protein interactions: turquoise—known from curated databases, pink—known from experimentally determined, green—predicted based on gene neighborhood, red—predicted based on gene fusion, blue—predicted based on gene co-occurrence, lime—inferred from text mining, black—revealed from co-expression evidence, light blue—inferred from protein homology. **C** Volcano plot depicting the impact of Senescence on the gene expression patterns in high glucose levels. Some of the DE gene names were omitted due to the overlapping labels. In blue: downregulated DE genes; in red: upregulated DE genes; in green: non-significant (ns) genes.

To gain insight into the molecular mechanisms driving the acquisition of a senescent phenotype following exposure to HG, we performed an RNA-seq assay on Ctr and Sen cells cultivated in presence/absence of HG culture medium (total number of samples = 12; number of samples in each cell type-medium condition group = 3). The differential expression (DE) analysis revealed that 48 genes were differentially expressed in Sen compared to Ctr cells as visualized on the volcano plot in Fig. 3A. 71% of DE genes were upregulated (*n* = 34) and 29% were downregulated (*n* = 14) in Sen cells, as listed in Table 1. Among the DE genes, 16 were previously associated with cellular senescence and are included in HAGR CellAge signature created basing on transcriptomic studies [16]. For all those entities, we observed a concordance in the direction of expression change between our experiment and HAGR database. 90% of the DE genes were present in SeneQuest database having evidence on their transcriptomic association with cellular senescence supported by a number of previously published studies [17]. Pathway enrichment analysis indicated the interferon alpha/beta signaling pathway (Reactome pathway ID: R-HSA-909733) as the most deregulated in senescent cells (22 query entities included corrected over-representation probability/FDR = 0.0005). Functional protein network analysis highlighted and further confirmed the role of DE genes in interferon Alpha/Beta signaling related to senescence in NG and detailed the protein-protein interactions between involved entities (Fig. 3B).

**Table 1:** Genes differentially expressed (adj.*p*-value < 0.10 and logFC > 1.5) in senescent cells compared to young in normal glucose levels.

#	Gene_ID	logFC	AveExpr	*t*	*P*.Value	Adj.P.Value	*B*	HGNC_ID	Description	HAGR_DB	SeneQuest_DB
1	ENSG00000092969	4.7564	3.6305	9.4846	0.0000	0.0118	5.6387	*TGFB2*	transforming growth factor beta 2 [Source:HGNC Symbol;Acc:HGNC:11768]	Over-expressed	U:5; D:1
2	ENSG00000005187	3.7810	1.5349	7.5726	0.0000	0.0254	3.1866	*ACSM3*	acyl-CoA synthetase medium chain family member 3 [Source:HGNC Symbol;Acc:HGNC:10522]	NA	U:2; D:0
3	ENSG00000105855	2.2509	3.8918	7.5565	0.0000	0.0254	4.0563	*ITGB8*	integrin subunit beta 8 [Source:HGNC Symbol;Acc:HGNC:6163]	NA	U:3; D:2
4	ENSG00000079616	−1.5695	5.6850	−7.5080	0.0000	0.0254	4.1239	*KIF22*	kinesin family member 22 [Source:HGNC Symbol;Acc:HGNC:6391]	Under-expressed	U:0; D:5
5	ENSG00000157601	3.5772	4.3326	7.4619	0.0000	0.0254	3.9681	*MX1*	MX dynamin like GTPase 1 [Source:HGNC Symbol;Acc:HGNC:7532]	Over-expressed	U:4; D:0
6	ENSG00000144554	−2.0280	4.3566	−7.4045	0.0000	0.0254	3.9519	*FANCD2*	FA complementation group D2 [Source:HGNC Symbol;Acc:HGNC:3585]	Under-expressed	U:2; D:7
7	ENSG00000185745	4.2406	0.9891	6.5060	0.0000	0.0606	1.9589	*IFIT1*	interferon induced protein with tetratricopeptide repeats 1 [Source:HGNC Symbol;Acc:HGNC:5407]	Over-expressed	U:2; D:1
8	ENSG00000144712	−1.8628	2.8348	−6.4925	0.0000	0.0606	2.7076	*CAND2*	cullin associated and neddylation dissociated 2 (putative) [Source:HGNC Symbol;Acc:HGNC:30689]	NA	U:1; D:1
9	ENSG00000138675	3.4917	2.7006	6.4308	0.0000	0.0606	2.4552	*FGF5*	fibroblast growth factor 5 [Source:HGNC Symbol;Acc:HGNC:3683]	NA	U:2; D:1
10	ENSG00000165072	4.8361	2.7871	6.4243	0.0000	0.0606	2.3163	*MAMDC2*	MAM domain containing 2 [Source:HGNC Symbol;Acc:HGNC:23673]	Over-expressed	U:2; D:0
11	ENSG00000141449	3.5485	2.3757	6.3410	0.0000	0.0623	2.3068	*GREB1L*	GREB1 like retinoic acid receptor coactivator [Source:HGNC Symbol;Acc:HGNC:31042]	NA	U:0; D:1
12	ENSG00000105426	1.7894	5.8019	6.2349	0.0000	0.0669	2.4319	*PTPRS*	protein tyrosine phosphatase receptor type S [Source:HGNC Symbol;Acc:HGNC:9681]	NA	U:1; D:1
13	ENSG00000105825	4.9582	6.2532	6.1551	0.0000	0.0696	2.3023	*TFPI2*	tissue factor pathway inhibitor 2 [Source:HGNC Symbol;Acc:HGNC:11761]	NA	U:3; D:2
14	ENSG00000067048	−5.2633	−0.8843	−6.0557	0.0001	0.0712	0.5763	*DDX3Y*	DEAD-box helicase 3 Y-linked [Source:HGNC Symbol;Acc:HGNC:2699]	NA	U:1; D:2
15	ENSG00000137193	−2.1836	3.1077	−6.0356	0.0001	0.0712	2.0950	*PIM1*	Pim-1 proto-oncogene, serine/threonine kinase [Source:HGNC Symbol;Acc:HGNC:8986]	Under-expressed	U:7; D:2
16	ENSG00000115896	2.0854	3.7362	6.0024	0.0001	0.0712	2.0557	*PLCL1*	phospholipase C like 1 (inactive) [Source:HGNC Symbol;Acc:HGNC:9063]	NA	U:1; D:1
17	ENSG00000149212	2.3423	3.4360	5.9643	0.0001	0.0712	1.9926	*SESN3*	sestrin 3 [Source:HGNC Symbol;Acc:HGNC:23060]	NA	U:2; D:0
18	ENSG00000167772	4.2680	4.5042	5.8609	0.0001	0.0743	1.8559	*ANGPTL4*	angiopoietin like 4 [Source:HGNC Symbol;Acc:HGNC:16039]	Over-expressed	U:7; D:1
19	ENSG00000172738	2.7465	3.2557	5.7940	0.0001	0.0743	1.7296	*TMEM217*	transmembrane protein 217 [Source:HGNC Symbol;Acc:HGNC:21238]	Over-expressed	U:1; D:0
20	ENSG00000067646	−4.9956	−2.4558	−5.7418	0.0001	0.0743	−0.5138	*ZFY*	zinc finger protein Y-linked [Source:HGNC Symbol;Acc:HGNC:12870]	NA	NA
21	ENSG00000138623	2.7519	1.4124	5.6953	0.0001	0.0743	1.3690	*SEMA7A*	semaphorin 7A (John Milton Hagen blood group) [Source:HGNC Symbol;Acc:HGNC:10741]	NA	U:2; D:2
22	ENSG00000066735	−3.0296	2.9913	−5.6590	0.0001	0.0743	1.5641	*KIF26A*	kinesin family member 26A [Source:HGNC Symbol;Acc:HGNC:20226]	NA	U:1; D:2
23	ENSG00000145242	4.5827	1.3860	5.6230	0.0001	0.0743	0.9377	*EPHA5*	EPH receptor A5 [Source:HGNC Symbol;Acc:HGNC:3389]	NA	NA
24	ENSG00000026103	1.8381	3.8246	5.6224	0.0001	0.0743	1.5320	*FAS*	Fas cell surface death receptor [Source:HGNC Symbol;Acc:HGNC:11920]	Over-expressed	U:4; D:1
25	ENSG00000189056	4.9245	3.4932	5.6037	0.0001	0.0743	1.4219	*RELN*	reelin [Source:HGNC Symbol;Acc:HGNC:9957]	NA	U:0; D:1
26	ENSG00000164484	3.0426	2.0086	5.6013	0.0001	0.0743	1.2714	*TMEM200A*	transmembrane protein 200A [Source:HGNC Symbol;Acc:HGNC:21075]	NA	U:1; D:0
27	ENSG00000165246	−5.5321	−2.3438	−5.5977	0.0001	0.0743	−0.5528	*NLGN4Y*	neuroligin 4 Y-linked [Source:HGNC Symbol;Acc:HGNC:15529]	NA	NA
28	ENSG00000119669	−1.7321	6.2914	−5.5917	0.0001	0.0743	1.4829	*IRF2BPL*	interferon regulatory factor 2 binding protein like [Source:HGNC Symbol;Acc:HGNC:14282]	NA	U:1; D:1
29	ENSG00000163071	1.5607	3.9695	5.5669	0.0001	0.0743	1.4508	*SPATA18*	spermatogenesis associated 18 [Source:HGNC Symbol;Acc:HGNC:29579]	Over-expressed	U:4; D:0
30	ENSG00000147459	1.5019	5.5256	5.5435	0.0001	0.0743	1.4137	*DOCK5*	dedicator of cytokinesis 5 [Source:HGNC Symbol;Acc:HGNC:23476]	NA	U:0; D:1
31	ENSG00000066248	5.8635	−0.3949	5.5192	0.0001	0.0743	−0.2762	*NGEF*	neuronal guanine nucleotide exchange factor [Source:HGNC Symbol;Acc:HGNC:7807]	NA	U:1; D:2
32	ENSG00000178726	2.0545	6.0271	5.5139	0.0001	0.0743	1.3684	*THBD*	thrombomodulin [Source:HGNC Symbol;Acc:HGNC:11784]	NA	U:5; D:2
33	ENSG00000163808	−1.9526	3.6393	−5.4162	0.0002	0.0800	1.2267	*KIF15*	kinesin family member 15 [Source:HGNC Symbol;Acc:HGNC:17273]	Under-expressed	U:0; D:5
34	ENSG00000170430	−2.9235	1.2568	−5.4140	0.0002	0.0800	0.9960	*MGMT*	O-6-methylguanine-DNA methyltransferase [Source:HGNC Symbol;Acc:HGNC:7059]	Under-expressed	NA
35	ENSG00000135074	1.5278	6.1145	5.4140	0.0002	0.0800	1.2119	*ADAM19*	ADAM metallopeptidase domain 19 [Source:HGNC Symbol;Acc:HGNC:197]	Over-expressed	U:2; D:0
36	ENSG00000089127	2.4090	3.2450	5.3647	0.0002	0.0821	1.1398	*OAS1*	2’-5’-oligoadenylate synthetase 1 [Source:HGNC Symbol;Acc:HGNC:8086]	NA	U:3; D:0
37	ENSG00000101311	2.0626	3.1539	5.3319	0.0002	0.0844	1.0810	*FERMT1*	FERM domain containing kindlin 1 [Source:HGNC Symbol;Acc:HGNC:15889]	NA	U:1; D:0
38	ENSG00000126709	3.8129	4.6387	5.2545	0.0002	0.0933	0.9805	*IFI6*	interferon alpha inducible protein 6 [Source:HGNC Symbol;Acc:HGNC:4054]	Over-expressed	U:4; D:1
39	ENSG00000184545	2.2951	3.4372	5.2215	0.0002	0.0961	0.9246	*DUSP8*	dual specificity phosphatase 8 [Source:HGNC Symbol;Acc:HGNC:3074]	NA	U:1; D:0
40	ENSG00000119514	2.4543	2.6776	5.1574	0.0002	0.0969	0.8039	*GALNT12*	polypeptide N-acetylgalactosaminyltransferase 12 [Source:HGNC Symbol;Acc:HGNC:19877]	NA	U:3; D:1
41	ENSG00000171346	2.1039	3.0590	5.1547	0.0002	0.0969	0.8167	*KRT15*	keratin 15 [Source:HGNC Symbol;Acc:HGNC:6421]	NA	U:1; D:0
42	ENSG00000185507	1.7958	3.9681	5.1542	0.0002	0.0969	0.8260	*IRF7*	interferon regulatory factor 7 [Source:HGNC Symbol;Acc:HGNC:6122]	NA	U:5; D:1
43	ENSG00000113368	−1.5113	6.3069	−5.1485	0.0002	0.0969	0.7895	*LMNB1*	lamin B1 [Source:HGNC Symbol;Acc:HGNC:6637]	Under-expressed	U:4; D:33
44	ENSG00000156113	3.7668	3.6239	5.1414	0.0002	0.0969	0.7966	*KCNMA1*	potassium calcium-activated channel subfamily M alpha 1 [Source:HGNC Symbol;Acc:HGNC:6284]	NA	U:1; D:1
45	ENSG00000154262	2.7302	3.8470	5.1331	0.0002	0.0969	0.7939	*ABCA6*	ATP binding cassette subfamily A member 6 [Source:HGNC Symbol;Acc:HGNC:36]	NA	U:1; D:1
46	ENSG00000186918	−2.3673	2.3481	−5.1207	0.0003	0.0969	0.7447	*ZNF395*	zinc finger protein 395 [Source:HGNC Symbol;Acc:HGNC:18737]	NA	U:1; D:3
47	ENSG00000115194	−2.5308	1.5416	−5.1075	0.0003	0.0970	0.6628	*SLC30A3*	solute carrier family 30 member 3 [Source:HGNC Symbol;Acc:HGNC:11014]	NA	U:0; D:1
48	ENSG00000147697	1.9285	3.1885	5.0923	0.0003	0.0975	0.7268	*GSDMC*	gasdermin C [Source:HGNC Symbol;Acc:HGNC:7151]	NA	NA

*logFC* log2 of fold change between two compared groups, *AveExpr* average log2 expression level for a gene across all compared samples, *t* modareted t-statistics, *P.Value and Adj.P.Value* nominal and corrected for multiple testing (with Benjamini and Hochberg’s method) *p*-values respectively, *B* log-odds that the gene is differentially expressed B-statistics, HGNC_ID, *Description* descriptive gene name, *HAGR_DB* if the gene is included in HAGR CellAge signature and eventually what is the direction of expression change assciated with senescence, *SeneQuest* if the gene is present in SeneQuest database and eventually the number of studies reporting it’s (U) up- and (D) down-regulated status.

We have identified 49 genes with significantly deregulated expression in Sen cells compared to Ctr in HG culture medium (Fig. 3C and Table 2). Similarly to senescence-related DE genes emerged from samples cultivated in normal glucose conditions, the entities were prevalently up-regulated (78%, *n* = 38) in senescence. Among identified deregulated genes, 11 items were comprised in HAGR CellAge signature and all of them had the direction of expression change concordant with the one reported in the database. 86% of the DE genes were additionally supported by the evidence reported in SeneQuest database. Pathway enrichment analysis did not identify any term that would reach statistical significance. Integrated protein network analysis revealed enrichment in proteins presenting conserved EGF-like domain (SMART Acc. Num.: SM00181; 6 query entities included; FDR = 0.0070). 32 of genes resulted as senescence-related in separated DE analysis were common between normal and high glucose levels and presented concordant direction of expression change in both conditions. This set of genes denotes a glucose-independent transcriptomic signal accompanying occurrence of senescence (Table 3, Set A). On the other hand, the unique sets of 16 and 17 genes differentially expressed in senescent compared to young cells in normal and high glucose levels respectively (Table 3, Set B and Set C), represent patterns stimulated in specific medium conditions. Multidimensional scaling (MDS) performed on RNA-seq counts of genes identified as Senescence-related and glucose-independent confirmed the potential of common gene set A to effectively capture the differences between senescent and young expression profiles (first MDS dimension explaining 93% of total variance observed in dataset) and to successfully separate these two cell cultures as visualized in Supplementary Fig. 1.

**Table 2:** Genes differentially expressed (adj.*p*-value < 0.10 and logFC > 1.5) in senescent cells compared to young in high glucose levels.

#	Gene_ID	logFC	AveExpr	*t*	*P*.Value	Adj.P.Value	*B*	HGNC_ID	Description	HAGR_DB	SeneQuest_DB
1	ENSG00000092969	4.1916	3.6305	8.6623	0.0000	0.0307	4.9546	*TGFB2*	transforming growth factor beta 2 [Source:HGNC Symbol;Acc:HGNC:11768]	Over-expressed	U:5; D:1
2	ENSG00000144712	−2.3175	2.8348	−7.1506	0.0000	0.0538	3.3569	*CAND2*	cullin associated and neddylation dissociated 2 (putative) [Source:HGNC Symbol;Acc:HGNC:30689]	NA	U:1; D:1
3	ENSG00000105855	2.2515	3.8918	7.6370	0.0000	0.0538	4.1361	*ITGB8*	integrin subunit beta 8 [Source:HGNC Symbol;Acc:HGNC:6163]	NA	U:3; D:2
4	ENSG00000165072	5.8573	2.7871	7.2416	0.0000	0.0538	3.0004	*MAMDC2*	MAM domain containing 2 [Source:HGNC Symbol;Acc:HGNC:23673]	Over-expressed	U:2; D:0
5	ENSG00000005187	3.2947	1.5349	6.8560	0.0000	0.0541	2.5490	*ACSM3*	acyl-CoA synthetase medium chain family member 3 [Source:HGNC Symbol;Acc:HGNC:10522]	NA	U:2; D:0
6	ENSG00000105825	5.7657	6.2532	6.9590	0.0000	0.0541	3.3400	*TFPI2*	tissue factor pathway inhibitor 2 [Source:HGNC Symbol;Acc:HGNC:11761]	NA	U:3; D:2
7	ENSG00000067048	−6.8101	−0.8843	−6.3715	0.0000	0.0596	−0.0209	*DDX3Y*	DEAD-box helicase 3 Y-linked [Source:HGNC Symbol;Acc:HGNC:2699]	NA	U:1; D:2
8	ENSG00000144554	−1.7887	4.3566	−6.4265	0.0000	0.0596	2.6770	*FANCD2*	FA complementation group D2 [Source:HGNC Symbol;Acc:HGNC:3585]	Under-expressed	U:2; D:7
9	ENSG00000049323	2.3622	7.9091	6.4720	0.0000	0.0596	2.7559	*LTBP1*	latent transforming growth factor beta binding protein 1 [Source:HGNC Symbol;Acc:HGNC:6714]	NA	U:3; D:2
10	ENSG00000141449	3.7801	2.3757	6.3966	0.0000	0.0596	2.2660	*GREB1L*	GREB1 like retinoic acid receptor coactivator [Source:HGNC Symbol;Acc:HGNC:31042]	NA	U:0; D:1
11	ENSG00000170430	−3.8995	1.2568	−6.3025	0.0000	0.0601	1.7769	*MGMT*	O-6-methylguanine-DNA methyltransferase [Source:HGNC Symbol;Acc:HGNC:7059]	Under-expressed	NA
12	ENSG00000147459	1.6732	5.5256	6.2033	0.0000	0.0601	2.3856	*DOCK5*	dedicator of cytokinesis 5 [Source:HGNC Symbol;Acc:HGNC:23476]	NA	U:0; D:1
13	ENSG00000138675	3.2701	2.7006	6.2275	0.0000	0.0601	2.2159	*FGF5*	fibroblast growth factor 5 [Source:HGNC Symbol;Acc:HGNC:3683]	NA	U:2; D:1
14	ENSG00000137193	−2.2256	3.1077	−5.9659	0.0001	0.0636	1.9719	*PIM1*	Pim-1 proto-oncogene, serine/threonine kinase [Source:HGNC Symbol;Acc:HGNC:8986]	Under-expressed	U:7; D:2
15	ENSG00000198825	1.7666	5.9339	5.9823	0.0001	0.0636	2.0688	*INPP5F*	inositol polyphosphate-5-phosphatase F [Source:HGNC Symbol;Acc:HGNC:17054]	NA	NA
16	ENSG00000115896	1.9091	3.7362	5.9657	0.0001	0.0636	2.0250	*PLCL1*	phospholipase C like 1 (inactive) [Source:HGNC Symbol;Acc:HGNC:9063]	NA	U:1; D:1
17	ENSG00000101311	2.3392	3.1539	6.0962	0.0001	0.0636	2.1481	*FERMT1*	FERM domain containing kindlin 1 [Source:HGNC Symbol;Acc:HGNC:15889]	NA	U:1; D:0
18	ENSG00000119514	3.0566	2.6776	6.0076	0.0001	0.0636	1.9177	*GALNT12*	polypeptide N-acetylgalactosaminyltransferase 12 [Source:HGNC Symbol;Acc:HGNC:19877]	NA	U:3; D:1
19	ENSG00000105426	1.6768	5.8019	5.9200	0.0001	0.0648	1.9777	*PTPRS*	protein tyrosine phosphatase receptor type S [Source:HGNC Symbol;Acc:HGNC:9681]	NA	U:1; D:1
20	ENSG00000138623	3.1386	1.4124	5.7901	0.0001	0.0721	1.2818	*SEMA7A*	semaphorin 7A (John Milton Hagen blood group) [Source:HGNC Symbol;Acc:HGNC:10741]	NA	U:2; D:2
21	ENSG00000026103	1.8876	3.8246	5.7141	0.0001	0.0744	1.6592	*FAS*	Fas cell surface death receptor [Source:HGNC Symbol;Acc:HGNC:11920]	Over-expressed	U:4; D:1
22	ENSG00000104368	2.6882	8.1704	5.7147	0.0001	0.0744	1.6748	*PLAT*	plasminogen activator, tissue type [Source:HGNC Symbol;Acc:HGNC:9051]	Over-expressed	U:6; D:3
23	ENSG00000066735	−3.4785	2.9913	−5.6444	0.0001	0.0767	1.4577	*KIF26A*	kinesin family member 26A [Source:HGNC Symbol;Acc:HGNC:20226]	NA	U:1; D:2
24	ENSG00000119669	−1.7622	6.2914	−5.6575	0.0001	0.0767	1.5866	*IRF2BPL*	interferon regulatory factor 2 binding protein like [Source:HGNC Symbol;Acc:HGNC:14282]	NA	U:1; D:1
25	ENSG00000184545	2.4739	3.4372	5.5964	0.0001	0.0790	1.4684	*DUSP8*	dual specificity phosphatase 8 [Source:HGNC Symbol;Acc:HGNC:3074]	NA	U:1; D:0
26	ENSG00000167772	3.7894	4.5042	5.5564	0.0001	0.0790	1.4305	*ANGPTL4*	angiopoietin like 4 [Source:HGNC Symbol;Acc:HGNC:16039]	Over-expressed	U:7; D:1
27	ENSG00000189056	5.0915	3.4932	5.5594	0.0001	0.0790	1.3161	*RELN*	reelin [Source:HGNC Symbol;Acc:HGNC:9957]	NA	U:0; D:1
28	ENSG00000135074	1.5376	6.1145	5.4864	0.0001	0.0827	1.3264	*ADAM19*	ADAM metallopeptidase domain 19 [Source:HGNC Symbol;Acc:HGNC:197]	Over-expressed	U:2; D:0
29	ENSG00000067646	−4.6118	−2.4558	−5.3053	0.0002	0.0840	−0.8754	*ZFY*	zinc finger protein Y-linked [Source:HGNC Symbol;Acc:HGNC:12870]	NA	NA
30	ENSG00000178726	1.9822	6.0271	5.3789	0.0002	0.0840	1.1622	*THBD*	thrombomodulin [Source:HGNC Symbol;Acc:HGNC:11784]	NA	U:5; D:2
31	ENSG00000124721	2.0016	2.1808	5.3036	0.0002	0.0840	0.9579	*DNAH8*	dynein axonemal heavy chain 8 [Source:HGNC Symbol;Acc:HGNC:2952]	NA	U:1; D:0
32	ENSG00000070404	2.1488	5.2819	5.3747	0.0002	0.0840	1.1596	*FSTL3*	follistatin like 3 [Source:HGNC Symbol;Acc:HGNC:3973]	NA	U:3; D:0
33	ENSG00000113356	2.1953	3.7905	5.3361	0.0002	0.0840	1.1008	*POLR3G*	RNA polymerase III subunit G [Source:HGNC Symbol;Acc:HGNC:30075]	NA	U:0; D:2
34	ENSG00000134242	2.4979	2.9072	5.3232	0.0002	0.0840	1.0453	*PTPN22*	protein tyrosine phosphatase non-receptor type 22 [Source:HGNC Symbol;Acc:HGNC:9652]	NA	U:0; D:2
35	ENSG00000164484	2.6151	2.0086	5.3420	0.0002	0.0840	0.9995	*TMEM200A*	transmembrane protein 200A [Source:HGNC Symbol;Acc:HGNC:21075]	NA	U:1; D:0
36	ENSG00000187688	3.7113	5.2333	5.3969	0.0002	0.0840	1.1976	*TRPV2*	transient receptor potential cation channel subfamily V member 2 [Source:HGNC Symbol;Acc:HGNC:18082]	Over-expressed	U:2; D:1
37	ENSG00000074211	4.8401	0.6851	5.4361	0.0002	0.0840	0.5009	*PPP2R2C*	protein phosphatase 2 regulatory subunit Bgamma [Source:HGNC Symbol;Acc:HGNC:9306]	NA	NA
38	ENSG00000218336	3.0841	7.0303	5.2565	0.0002	0.0886	0.9753	*TENM3*	teneurin transmembrane protein 3 [Source:HGNC Symbol;Acc:HGNC:29944]	NA	U:2; D:0
39	ENSG00000147697	1.9896	3.1885	5.2279	0.0002	0.0907	0.9258	*GSDMC*	gasdermin C [Source:HGNC Symbol;Acc:HGNC:7151]	NA	NA
40	ENSG00000161267	−2.0363	2.0796	−5.1577	0.0002	0.0938	0.7535	*BDH1*	3-hydroxybutyrate dehydrogenase 1 [Source:HGNC Symbol;Acc:HGNC:1027]	NA	U:1; D:0
41	ENSG00000186638	−1.8549	3.0403	−5.1529	0.0002	0.0938	0.8068	*KIF24*	kinesin family member 24 [Source:HGNC Symbol;Acc:HGNC:19916]	NA	U:0; D:4
42	ENSG00000149212	1.9610	3.4360	5.1618	0.0002	0.0938	0.8341	*SESN3*	sestrin 3 [Source:HGNC Symbol;Acc:HGNC:23060]	NA	U:2; D:0
43	ENSG00000163814	2.3223	4.9574	5.1832	0.0002	0.0938	0.8673	*CDCP1*	CUB domain containing protein 1 [Source:HGNC Symbol;Acc:HGNC:24357]	NA	U:1; D:1
44	ENSG00000115884	3.0668	4.3592	5.1128	0.0003	0.0962	0.7627	*SDC1*	syndecan 1 [Source:HGNC Symbol;Acc:HGNC:10658]	NA	U:5; D:1
45	ENSG00000135451	−1.6411	4.3455	−5.0707	0.0003	0.0962	0.6932	*TROAP*	trophinin associated protein [Source:HGNC Symbol;Acc:HGNC:12327]	Under-expressed	U:0; D:6
46	ENSG00000197093	1.8652	3.4063	5.0652	0.0003	0.0962	0.6865	*GAL3ST4*	galactose-3-O-sulfotransferase 4 [Source:HGNC Symbol;Acc:HGNC:24145]	NA	U:2; D:0
47	ENSG00000171346	2.0823	3.0590	5.0816	0.0003	0.0962	0.7013	*KRT15*	keratin 15 [Source:HGNC Symbol;Acc:HGNC:6421]	NA	U:1; D:0
48	ENSG00000145242	3.6229	1.3860	5.0949	0.0003	0.0962	0.4706	*EPHA5*	EPH receptor A5 [Source:HGNC Symbol;Acc:HGNC:3389]	NA	NA
49	ENSG00000133800	2.5900	5.8922	5.0322	0.0003	0.0998	0.6211	*LYVE1*	lymphatic vessel endothelial hyaluronan receptor 1 [Source:HGNC Symbol;Acc:HGNC:14687]	NA	NA

*logFC* log2 of fold change between two compared groups, *AveExpr* average log2 expression level for a gene across all compared samples, *t* modareted t-statistics, *P.Value and Adj.P.Value* nominal and corrected for multiple testing (with Benjamini and Hochberg’s method) p-values, respectively, *B* log-odds that the gene is differentially expressed, B-statistics, HGNC_ID, *Description* descriptive gene name, *HAGR_DB* if the gene is included in HAGR CellAge signature and eventually what is the direction of expression change assciated with senescence, *SeneQuest* if the gene is present in SeneQuest database and eventually the number of studies reporting it’s (U) up- and (D) down-regulated status.

**Table 3:** Lists of genes differentially expressed (adj.*p*-value < 0.10 and logFC > 1.5) in senescent cells compared to young: (A) commonly in normal and high glucose levels; (B) exclusively in normal glucose levels; (C) exclusively in high glucose levels.

Differential expression	Set A	Set B	Set C
Upregulated	ACSM3	ABCA6	CDCP1
	ADAM19	IFI6	DNAH8
	ANGPTL4	IFIT1	FSTL3
	DOCK5	IRF7	GAL3ST4
	DUSP8	KCNMA1	INPP5F
	EPHA5	MX1	LTBP1
	FAS	NGEF	LYVE1
	FERMT1	OAS1	PLAT
	FGF5	SPATA18	POLR3G
	GALNT12	TMEM217	PPP2R2C
	GREB1L		PTPN22
	GSDMC		SDC1
	ITGB8		TENM3
	KRT15		TRPV2
	MAMDC2		
	PLCL1		
	PTPRS		
	RELN		
	SEMA7A		
	SESN3		
	TFPI2		
	TGFB2		
	THBD		
	TMEM200A		
Downregulated	CAND2	KIF15	BDH1
	DDX3Y	KIF22	KIF24
	IRF2BPL	LMNB1	TROAP
	KIF26A	NLGN4Y	
	MGMT	SLC30A3	
	PIM1	ZNF395	
	ZFY		

### HG and senescence are associated with the accrual of cytoplasmatic nucleic acids in endothelial cells

**Fig. 4:**
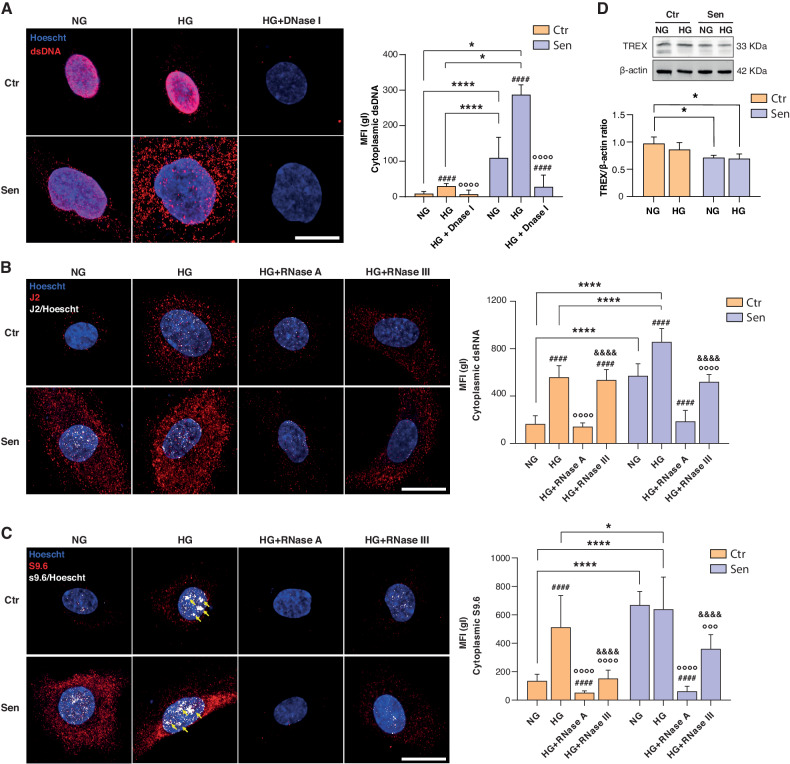
High glucose-treated cells and senescent cells accumulate cytoplasmic self-derived nucleic acids. **A** Representative images of nucleic and cytosolic double-stranded DNA (dsDNA) in HUVECs marked with anti-dsDNA antibody. Nuclei were labeled with Hoechst 33342 dyes (blue fluorescence). The specificity of the antibody was tested by using DNase I activity. Scale bar: 10 µm. **B** Anti-double-stranded RNA antibody (J2) was used to label dsRNA in HUVECs in presence/absence of high glucose treatment. RNase A and RNase III. White areas indicate co-localization of J2 antibody and Hoechst 33342 dyes. Scale bar: 10 µm. **C** S9.6. #, vs NG; °, vs. HG; &, vs HG+RNase A. White areas and yellow arrows indicate co-localization of S9.6 antibody and Hoechst 33342 dyes. Scale bar: 10 µm. **D** Western blot and densitometric analysis of TREX1. β-actin was used as a housekeeping protein. Data are mean ± SD of three independent experiments. *p < 0.05; **p < 0.01; ***p < 0.001; ****, ^####^, ^&&&&^ and °°°°*p* < 0.0001 for paired (HG vs. NG and DNase I/RNase III vs. HG) and unpaired (Sen vs. Ctr) *t* tests.

Based on the observation of a functional enrichment of the interferon alpha/beta signaling in Sen cells, we focused our attention on the role of self-derived nucleic acids, which could trigger a type I interferon response by binding DNA and RNA cytosolic sensors. The misplacement of DNA and dsRNA into the cytoplasm of senescent cells has been previously reported [4, 5]. By immunofluorescence, we assessed the abundance and subcellular localization of dsDNA, dsRNA, and RNA:DNA hybrids in Ctr and Sen cells, cultured in presence or absence of HG. We observed a significant increase in the levels of cytosolic dsDNA (12-fold) and dsRNA (3.5-fold) in Sen cells (Fig. 4A, B). Moreover, HG exposure further increased the burden of cytosolic dsDNA and dsRNA (Fig. 4A, B) in both Ctr and Sen cells. Similarly, a 5-fold higher level of putative RNA:DNA hybrids, measured using an anti-S9.6 Ab, was shown in Sen cells, and HG-exposed Ctr cells (Fig. 4C). Since the S9.6 Ab detects both RNA:DNA hybrids and dsRNA [18], cells were treated with RNase III, an enzyme that specifically degrades dsRNA. Treatment with RNase III did not decrease the fluorescence intensity of the S9.6 antibody, confirming that a substantial fraction of the cytoplasmic signal in HG-exposed Ctr cells is attributable to RNA:DNA hybrids (Fig. 4C). Conversely, treatment with RNase A was associated with an almost complete abrogation of the S9.6 signal (see Fig. 4B and C, last panel). Since cytosolic double-stranded nucleic acids and RNA:DNA hybrids were significantly increased in Sen cells, we analyzed the expression of three-prime Repair EXonuclease 1 (TREX1), a nonprocessive 3’ → 5’ exonuclease that is involved in the nucleic acid homeostasis in the cytoplasm [19–21]. We observed a significant reduction of TREX1 expression in Sen *vs* Ctr cells (Fig. 4D), in line with our previous observation that also RNaseH2 is decreased in senescent HUVECs [22]. On the other hand, the expression of TREX1 was not affected by exposure to HG. Hence the data suggest that the increased burden of cytosolic nucleic acids observed in Sen cells and in cells treated with HG is attributable, at least in part, to a mismatch between the rate of nucleic acid accumulation and the activity of nucleic acid degrading enzymes [2].

### HG- and senescence-associated accumulation of self-derived cytosolic nucleic acids induces the activation of RNA-sensing pathways

To elucidate the downstream effects of self-derived cytosolic nucleic acids in senescence and HG conditions, we analyzed both the DNA-sensing pathway, including TLR9, cyclic GMP–AMP synthase (cGAS), interferon (IFN)-inducible protein (IFI-16), and stimulator of interferon genes (STING), and the RNA-sensing pathway, i.e., melanoma differentiation-associated protein 5 (MDA5) and RIG-I-like receptor (RIG-I). Worth mentioning, TLR9 is able to sense also RNA:DNA hybrids [23]. In addition, we analyzed the expression of the downstream transcription factors interferon regulatory factor 3 (IRF3) and 7 (IRF7), which are engaged by both pathways.

**Fig. 5:**
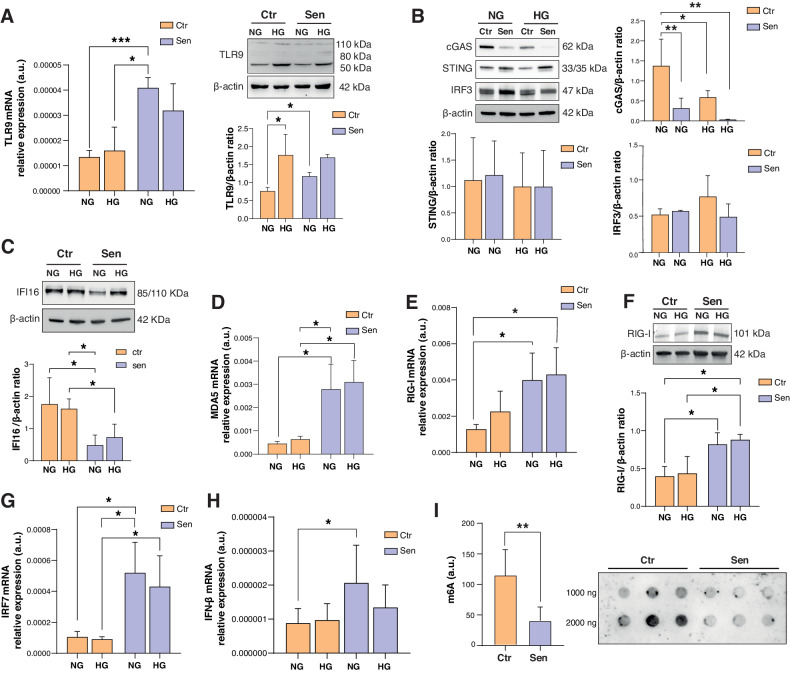
Cytosolic RNA sensors are upregulated in high glucose-treated and senescent HUVECs. **A** mRNA relative expression and western blot with densitometric analysis of TLR9 in Ctr and Sen cells. Protein expression values are reported as TLR9/β-actin ratio. **B** Western blot and densitometric analysis of cGAS, STING, and IRF3 in Ctr and Sen cells. β-actin was used as housekeeping protein. **C** Western blot and densitometric analysis of IFI16 in Ctr and Sen cells. β-actin was used as housekeeping protein. **D** MDA5 mRNA relative expression in arbitrary units (a.u.) in Ctr and Sen cells obtained through Real Time PCR. Data were normalized using β-actin as internal control. **E** RIG-I mRNA relative expression in arbitrary units (a.u.) in Ctr and Sen HUVECs, β-actin was used as internal control. **F** Western blot with densitometric analysis of RIG-I in Ctr and Sen cells. Protein expression values are reported as RIG-I/β-actin ratio. **G** IRF7 relative expression in arbitrary units (a.u.) in Ctr and Sen cells obtained through Real Time PCR. Data were normalized using β-actin as internal control. **H** IFN-β1 relative expression in arbitrary units (a.u.) in Ctr and Sen cells obtained through Real Time PCR. Data were normalized using β-actin as internal control. **I** Representative dot blot of total RNA isolated from Ctr and Sen cells. 1-2 µg of RNA was probed with the antibody against N6-methyladenosine (m6A) at 1:1000 dilution in TBS 3% BSA. Data are mean ± SD of three independent experiments. *t test *p* < 0.05; **t test *p* < 0.01.

Regarding the DNA-sensing pathway, we observed a significantly increased protein expression of TLR9 in Sen *vs* Ctr and in HG-Ctr *vs* Ctr cells (Fig. 5A). Surprisingly, we observed that both senescence and HG-induced a significant downregulation of cGAS, with Sen cells in HG showing the lowest expression (Fig. 5B). On the contrary, IFI-16 was downregulated in Sen cells but not in HG-treated Ctr cells (Fig. 5C), while STING levels were not significantly modulated by either senescence or HG (Fig. 5B). At variance, the self-RNA sensing pathway, both MDA5 (Fig. 5D) and RIG-I (Fig. 5E, F) were overexpressed in Sen *vs* Ctr, both in NG and HG conditions.

Overall, the cytosolic RNA sensors appear upregulated in Sen *vs* Ctr both in NG and HG, whereas the cytosolic DNA and RNA:DNA hybrid sensors were under-expressed in Sen compared to Ctr. This result is in line with the observation that cytoplasmic RNA was more abundant than DNA in Sen *vs* Ctr. The interferon regulatory factor IRF7 was significantly upregulated in Sen vs Ctr (Fig. 5G), whereas IRF3 expression was not changed in Sen cells (Fig. 5B). The increased expression of IRF7 was paralleled by a concomitant increase in the transcription of the downstream IFN-β1 mRNA in Sen cells (Fig. 5H), albeit not accompanied by an increased IFN-β1 release in conditioned medium (data not shown). Surprisingly, no differences in the expression of either IRF7 or IFN-β1 were observed under HG conditions compared to NG (Fig. 5G, H).

At variance, IL-6 was significantly overexpressed and released in Sen *vs* Ctr both in NG and HG conditions (Fig. 2C-D). Overall, the increased burden of cytosolic self-derived DNA and RNA observed in senescent HUVECs, both in NG and HG, induces an increased synthesis and release of IL-6, without a concomitant increase of type I IFN.

To better understand the possible causes of the reduced ability of senescent cells to produce type I interferon we investigated the most prevalent RNA chemical modification, the methylation of adenosine A at the position of N6 (m6A) modification that can affect dsRNA sensing and activation of the downstream NF-κB and IFN-I pathways [24]. A dot blot demonstrated that Sen endothelial cells are characterized by significant RNA hypomethylation compared to Ctr cells (Fig. 5I).

### Differential inflammatory and type I IFN responses triggered by exogenous double-stranded RNA in HUVECs

**Fig. 6:**
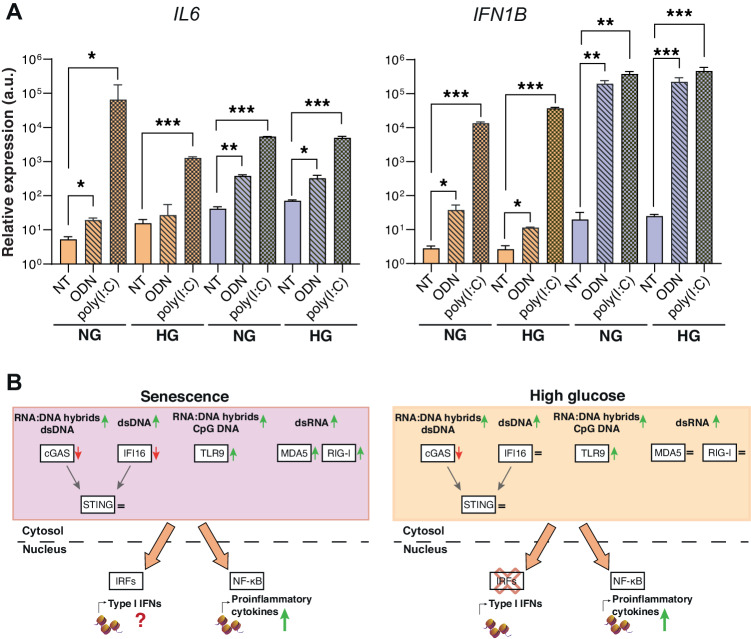
Exogenous DNA and RNA induce IL-6 and IFN-β1 expression. **A** IL-6 and IFN-β1 relative expression of Ctr and Sen cells in NG and HG condition. HUVECs were treated with 1 µM of ODN and poly I:C for 24 h. Data were normalized using β-actin as internal control and represented as ODN or poly I:C/NT ratio. *t test *p* < 0.05; **t test *p* < 0.01; ***t test *p* < 0.001; ****t test *p* < 0.0001. **B** Summary of the differential regulation of cytosolic DNA and RNA sensing pathways in HUVECs under replicative senescence and high glucose conditions. ↑, upregulated; ↓, downregulated.

To better investigate whether the phenomenon described above is applicable also to exogenous nucleic acids, that may mimic the response to viral infection, we exposed cells to the synthetic DNA molecule CpG ODN and polyinosinic:polycytidylic acid (poly I:C), a synthetic analog of the double-stranded RNA, considered as “natural” stimulants of TLR9 and TLR3, respectively [25, 26]. Sen cells showed significantly increased expression of IL-6 and IFN-β1 compared to untreated cells (NT) when stimulated with both these molecules, suggesting that Sen can trigger IFN-β1expression when stimulated by exogenous nucleic acids (Fig. 6A). In Fig. 6B, we summarize the modulation of nucleic acid sensing pathways in replicative senescence and in high glucose-exposed senescence ECs.

### DNA fragments are loaded into EVs released by senescent HUVECs

**Fig. 7:**
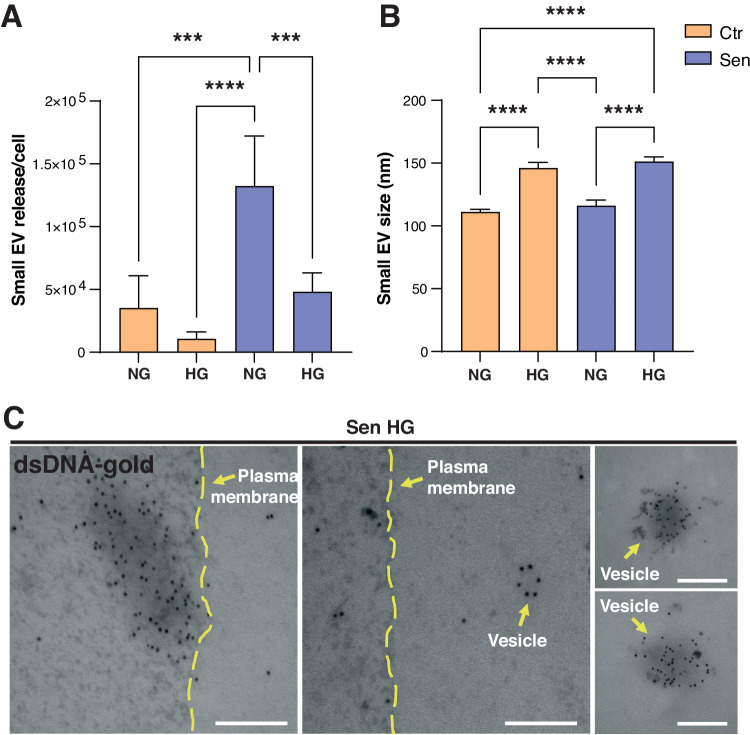
Senescent HUVECs release extracellular vesicles loaded with dsDNA. **A** Number per cell and (**B**) size of small EVs released in Ctr and Sen in the absence or in the presence of HG determined by NTA. **C** Representative TEM images of dsDNA in EVs of Sen cells in HG. Scale bars: 100 nm. ***t test *p* < 0.001; ****t test *p* < 0.0001.

Since replicative senescence and HG are accompanied by an increased amount of cytosolic DNA, which is linked with minimal activation of the self-DNA sensing pathways, we tested whether DNA fragments could be loaded inside EVs released by endothelial cells under extreme stress conditions. First, we extensively characterized EVs released by HUVECs by flow cytometry and Nanoparticle tracking analysis (NTA). The MACSPlex exosome protein marker analysis of isolated EVs confirmed the expression of the exosome markers CD9, CD63, and CD81, for all the experimental conditions (Supplementary Figure 2A). We confirmed our previous observation that, under NG conditions, Sen cells release a higher number of small EVs (Fig. 7A) [27]. Notably, exposure to HG significantly reduced the number of small EVs released by both Ctr and Sen cells, whilst increasing their size (Fig. 7B). Representative NTA plots are reported in Supplementary Fig. 2B. As shown in Fig. 7C, dsDNA was detectable in EVs released by SEN in HG, confirming that stressed cells can load nucleic acid inside EVs released in the milieu.

## Discussion

Increasing evidence is highlighting that “unmasked”, misprocessed, or misplaced host-derived RNA or DNA molecules can be recognized by cytosolic sensors, thus triggering antiviral host defenses or causing inflammation [28].

These mechanisms are understudied in the pathophysiology of endothelial dysfunction which, in turn, is central to the development of the most common age-related diseases. Endothelial dysfunction can be triggered by different mechanisms, including cellular senescence and high glucose exposure, and represents a major determinant of inflammaging [29]. In this framework, we aimed to investigate the effects of senescence and high glucose treatment on the accumulation of endogenous cytoplasmic nucleic acids, and on their ability to activate specific downstream pathways in human endothelial cells.

We demonstrated that endothelial cell replicative senescence is characterized by an increased burden of cytoplasmic nucleic acids and that a high glucose treatment can induce some features of senescence, such as DNA damage, telomere attrition, increased proinflammatory cytokine release, and increased burden of cytoplasmic misplaced nucleic acids, in young/functional endothelial cells. Our experimental conditions, a longtime cell exposure to HG (7 days), could mirror a chronic hyperglycemic condition more than an acute glucose-induced stress (i.e., 24-h HG) [30]. In this regard, we previously showed that such exposure was sufficient to induce senescence and the SASP in endothelial cells [13].

It has been previously reported that cellular senescence is accompanied by the accrual of DNA fragments in the cytoplasm, both due to increased DNA damage and enhanced permeability of the nuclear envelope following lamin B1 downregulation. The removal of such DNA fragments relies on the activity of cytoplasmic DNAses, including TREX1, and on the functional integrity of the autophagic/lysosomal machinery. Here, we confirmed the reduced TREX1 expression (DNase III) previously observed in senescent cells, strongly supports the increased amounts of cytoplasmic dsDNA [2]. Similarly, we reported increased levels of dsRNA and RNA:DNA hybrids in the cytoplasm of senescent and HG-treated ECs. One mechanism leading to the accumulation of cytoplasmic dsRNA and RNA:DNA hybrids during cellular senescence is related to the relocalization of sirtuins to areas of DNA double-strand break, causing the loss of heterochromatin structure and ultimately leading to the transcription of retrotransposable elements [31]. In this regard, we previously demonstrated a higher abundance of the RNA and complementary DNA sequences of two among the major retrotransposable elements, i.e., Alu and LINE1, in senescent ECs [32]. The increased presence of cytoplasmic RNA:DNA hybrids in senescent and HG-treated ECs corroborates our previous observation of reduced expression of RNase H2, the enzyme that specifically degrades the RNA moiety of RNA:DNA hybrids [22]. Our observations should be contextualized in the framework of the accumulation of endogenous/self, misplaced, or altered molecules in senescent cells that have been collectively designated as “garbaging” [33], a term coined to describe aging paralleled and mostly due to the accumulation of molecules that trigger inflammation and interferon response.

To better investigate the biological effects of cytoplasmic nucleic acid accumulation in senescent cells, we performed a transcriptomic profile to identify gene expression patterns associated with senescence and HG exposure. Notably, a significant proportion of DE genes in both senescent and HG conditions were comprised in the HAGR CellAge and SeneQuest signatures and all of them had the direction of expression change concordant with the one reported in the database. Interestingly, we reported two distinct sets of transcripts that showed a differential regulation in senescent cells according to exposure to normal or high glucose conditions. This result, which deserves further investigation, supports the ability of high glucose to reshape, at least at the transcriptional level, the senescence phenotype.

Starting from the observation that the type I IFN pathway was the most deregulated pathway in senescent cells, we evaluated the expression of selected cytosolic sensors that have been implicated in the recognition of endogenous DNA and RNA, such as TLR9, cGAS-STING and RIG-I [34]. TLR9 is localized in endosomes, and it is preferentially expressed in immune cells where it scrutinizes extracellular material taken up by receptor-mediated endo-phagocytosis. However, TLR9 can also recognize self-DNA, including the mtDNA and RNA:DNA hybrids [35, 36]. Interestingly, TLR9, which was significantly hyper-expressed in senescent cells, is activated by DNA containing unmethylated CpG motifs [36], and replicative senescence is accompanied by a global hypomethylation of DNA [27]. cGAS-STING is ubiquitously distributed in every cell type, patrolling the cytosol for the presence of viral or self-DNA extruded from inner compartments such as mitochondria and nuclei. TLR9 was significantly hyper-expressed in senescent cells and, albeit only at the protein level, also in HUVECs exposed to HG. In contrast with previous evidence [37], in our cellular model, the accumulation of cytoplasmic DNA observed during senescence was not paralleled by increased expression of the DNA sensor cGAS. The mechanisms underlying the non-linear relationship between the increased burden of self-derived nucleic acids and the differential expression of nucleic acid sensors remain to be elucidated.

Interestingly, we were able to detect the presence of dsDNA in EVs released by senescent ECs. In line with the notion that EV secretion could represent an alternative way to alleviate intracellular stress when the conventional recycling pathways, including autophagy and lysosomal degradation, are compromised [38], we can hypothesize that senescent ECs release a higher number of EVs containing DNA fragments to prevent excessive cGAS activation. Here, we showed that exposure to high glucose also induces the release of larger EVs. Notably, a previous study dissecting the subpopulations of EVs derived from ECs stimulated with TNF-α revealed that the pro-inflammatory phenotype of parent cells is more reflected in the larger EV subfraction, which is more able to propagate inflammation in recipient cells [39].

Regarding the RNA sensor RIG-I, it is critical for sensing viral RNA in the cytoplasm of mammalian host cells [40]. However, recent studies have revealed that both viral and host-derived RNAs can trigger RIG-I activation; this can lead to an effective antiviral response but also to immune diseases, especially when RIG-I-like receptor activities are uncontrolled, as expected when an increased burden of self RNA and DNA occurs [28]. We observed a significantly increased expression of RIG-I in senescent cells, but not in young cells treated with HG, despite the accrual of dsRNA. To better characterize the RIG-I sensing pathway, we also analyzed some interferon regulatory factors (IRFs), transcription factors that play pivotal roles in type IFN I production. As expected, a significantly increased expression of IRF7 was observed in senescent cells, as well as in young cells treated with HG, suggesting that the axis RIG-I/IRF7 is engaged in endothelial cells during senescence and under glucose-induced stress. Surprisingly, the activation of the RIG-I pathway in senescent cells was not accompanied by increased IFN-β1 synthesis, whereas IL-6 and IL-8 were hyper-expressed. These results suggest that endogenous dsRNA or RNA:DNA hybrids accumulated in Sen cells are active in stimulating proinflammatory pathways but do not converge on type IFN-I increased expression. Indeed, while we observed an upregulation of IFN-β mRNA, we were unable to detect a significant concentration of IFN-β in conditioned media. This hypothesis is reinforced by our observation that when senescent cells were treated with a strong exogenous stimulus, i.e. Poly I:C, they showed a sustained type IFN-I response, independently from HG condition. Overall endothelial senescent cells seem more prone to activate proinflammatory pathways in response to endogenous misplaced nucleic acids than IFN-I pathway, even if they maintain the ability to respond to exogenous stimuli. Here, we provided some evidence that the proinflammatory response observed in senescent cells in association with an increased burden of endogenous cytoplasmic nucleic acids can be due, at least in part, to the loss of one of the most prevalent RNA chemical modifications, i.e. the methylation of adenosine A at the position of N6 (m6A), that can foster an innate immune response and promote IFN-I pathway activation upon activation of the RIG-I RNA sensor [41, 42]. In our experimental model, we did not observe a sustained type I IFN response, which was previously reported upon blockade of the m6A methylation machinery in hematopoietic stem cells [42]. Importantly, we were able to confirm previous findings providing a mechanistic explanation that sensing of dsRNA by RIG-I resulted in increased proinflammatory cytokine expression in senescent HUVECs [43].

Over the past decades, ample progress has been made toward defining the molecular features that allow innate immune sensors to discriminate between endogenous and pathogen-derived RNA or DNA [44, 45]. Whereas cytoplasmatic exogenous nucleic acid recognition can mount an antiviral response and/or a proinflammatory response, intriguing research highlighted some deleterious effects on the host organism when endogenous cytoplasmic nucleic acids are detected [46, 47]. In this case, recognition of “self” represents an indirect mechanism of pathogen detection wherein the mammalian host senses viral manipulation of host RNA/DNA metabolism, or virus-induced changes in the subcellular localization of host nucleic acids [48].

Here, we provided evidence that, in the absence of viral infection, senescent cells and /or cells treated with high glucose can activate a proinflammatory response in association with an increased burden of cytoplasmic nucleic acids, and this mechanism could contribute perpetuating the deleterious systemic effects of the SASP in vivo. Owing to their ability to trigger innate immune responses, DNA and other nucleic acids localized in the cell cytoplasm or at the extracellular level should be regarded as major contributors to inflammaging and, therefore, as an emerging mechanism explaining the development of chronic age-related diseases. Further research is warranted to clarify the role of endothelial dysfunction induced by the accrual of self-nucleic acids in the aging process and the development and progression of human ARDs.

## Materials and methods

### Cell lines and culture

Human Umbilical Vein Endothelial Cells (HUVECs) were purchased from Clonetics (Lonza, Switzerland) as a pool of three donors and were cultured in EGM-2, endothelial growth medium (EBM-2, CC-3162, Lonza), supplemented with SingleQuot Bullet Kit (CC-4176, Lonza). Briefly, fresh cells were seeded in T25 flasks (Corning Costar, Sigma Aldrich, USA) and sub-cultured when they reached 70–80% confluence at a density of 5000/cm^2^ in a humidified atmosphere of 5% CO_2_ at 37 °C. The medium was changed at 48 h intervals. All cells tested were negative for mycoplasma infection. The passages of trypsinization and cell count are repeated until replicative Senescence, in this way the replication Senescence curve is obtained. Each step calculates population doubling (PD) which is given by the formula: (log_10_ F – log_10_ I)/log_10_2 where I represents the number of plated cells and F the number of viable cells counted the following passage and cumulative population doubling (CPD) was calculated as the sum of all PD changes. When the value of I is equal to or less than the value of F, the cells are in replicative block. In the experiments carried out, replicative senescence was obtained from 18 replication cycles. After reaching confluence, low-passage control HUVECs and senescent HUVECs were stimulated with 25 mM D-glucose (high glucose, HG) or normal EGM-2 (5.5 mM glucose, CTR) for a week. For CpG ODN (cat. no. tlrl-ttagc, InvivoGen, San Diego, CA, USA) and poly I:C (cat. no. P1530, Sigma Aldrich, Germany) treatment, HUVECs were incubated for 24 h in NG and HG medium at a concentration of 1uM. The proper HG and NG conditions have been tested in parallel.

### Lysosomal senescence associated–β-galactosidase activity

The percentage of Senescent cells has been evaluated using Senescence Detection Kit (cat. no. K320, BioVision Inc., USA), according to the manufacturer’s instructions. Cells were divided into control (Ctr; SA-β-Gal < 10%) and senescent (Sen; SA-β-Gal > 80%).

### Telomere length

Telomere length was analysed by quantitative PCR using Cawthon’s method [49]. Genomic DNA was isolated from each cell condition using QIAamp DNA Blood Kits (cat. no. 51104, Qiagen, Germany).

### mRNA expression analysis

Total RNA from young and Senescent HUVECs in normal and high glucose conditions were isolated using Norgen Biotek Kit (cat. no. 37500, Norgen Biotek Corporation, Canada), according to the manufacturer’s instructions. RNA was stored at −80 °C until use. mRNA expression was assessed as previously described [27]. Briefly 1 μg of purified RNA is reverse-transcribed following the protocol PrimeScript™ RT Reagent Kit with gDNA Eraser (cat. no. RR047A, Takara Bio Inc. USA). Real-time PCR reactions were conducted in a Rotor Gene Q 5plex HRM apparatus (Qiagen, Germany) in a 10 μl total reaction volume using TB Green Premix Ex Taq II (Tli Rnase H Plus, cat. no. RR420A, Takara Bio Inc.) according to the manufacturer’s instructions. The mRNA expression of the genes of interest was calculated using β-actin as reference gene, using the 2^−ΔCt^ method.

The primers sequences (written 5ʹ-3ʹ) were:

p16, Fw: CATAGATGCCGCGGAAGGT, Rv: CTAAGTTTCCCGAGGTTTCTCAGA;

IL-1β, Fw: CCAGCTACGAATCTCCGACC, Rv: TGGGGTGGAAAGGTTTGGA;

IL-6, Fw: CCAGCTACGAATCTCCGACC, Rv: CATGGCCACAACAATGACG;

IL-8, Fw: TCTGCAGCTCTGTGTGTGAAGG, Rv: TGGGGTGGAAAGGTTTGGA;

IFN1B, Fw: TGCTCTCCTGTTGTGCTTCT; Rv: CATAGATGGTCAATGCGGCG

β-actin, Fw: TGCTATCCCTGTACGCCTCT, Rv: GTGGTGGTGAAGCTGTAGCC;

Final primer concentration was 200 nM.

### Protein extraction and immunoblotting

For immunoblotting, cells were lysed in RIPA buffer (150 mM NaCl, 10 mM Tris, pH 7.2, 0.1% SDS, 1.0% Triton X-100, 5 mM EDTA, pH 8.0) containing protease and phosphatase inhibitors (Roche Applied Science, Indianapolis, IN, USA), separated by SDS-PAGE, transferred to nitrocellulose membranes (Whatman, Germany) and blocked in EveryBlot Blocking Buffer (cat. no. 12010020, Bio-Rad Laboratories, Hercules, CA, USA). Membranes were blotted with primary antibodies followed by horseradish peroxidase (HRP)-conjugated secondary antibodies antibody (Vector, USA). Primary antibodies targeting cGAS (1:1000; #15102), STING (1:1000; #13647), TREX1 (1:1000; #15107) were from Cell Signaling. p16(Ink4a) (1:200; sc-377412) and RIG-I (1:100; sc-376845) were from Santa Cruz Biotechnology, SIRT1 (1:1000; ab12193) was from Abcam, IRF-3 (1:2000; GTX133768) and TLR-9 (1:1000; NBP2-24729) from GeneTex and Novus Biologicals respectively. Immunoreactive proteins were visualized using Clarity Western ECL Substrate (cat. no. 1705060S, Bio-Rad Laboratories, Hercules, CA, USA) and quantified using ImageJ2 software [50]. Membranes were incubated with anti β-actin (1:3000; #3700, Cell Signalling Technology) as normalizer. Full length uncropped original western blots are available as a Supplemental Material file.

### Fluorescent in situ hybridization (FISH)

A probe was used to detect the presence of telomere DNA at the cytoplasmic level, capable of tying the CCATTG telomere sequence and emitting fluorescence, Telomere PNA Fisch kit/Fitc (Dako, cat. no. K5325, Agilent, CA, USA). The cells were fixed on slides by methanol passages for 10 min and acetone for 1 minute. The slides were treated following the procedure recommended by the product datasheet.

### Immunofluorescence

Controls and high glucose-treated cells were seeded at a density of 8000/cm^2^ on autoclaved polylysine-coated coverslips. Cells were washed twice with PBS and then fixed in paraformaldehyde 4% (PFA) in PBS for 1 h at 4 °C. After three washes in blocking buffer (5% FBS, 0.3% Triton X-100 in PBS), cells were incubated with anti-Phospho-Histone H2A.X (Ser139) antibody (1:200, #9718, Cell Signalling Technology) and anti-DNA-RNA hybrid antibody (cloneS9.6, 1:100; cat. no. MABE1095, MerckMillipore, CA, USA) in blocking buffer overnight at 4 °C. For anti-dsDNA cells were incubated with 0.5% Triton X-100 solution in PBS 1X for 10 min with rocking. After three PBS washes for 5 min, cells were incubated with 1% BSA in PBST (0.01% tween 20 in PBS) for 30 min. The primary antibody (1:100, cat. no. ab27156, clone 35I9 DNA, Abcam, UK) was diluted in 1% BSA in PBST in a humidified chamber for 1 h overnight at 4 °C. For anti-dsRNA (J2; 1:200; cat. no. 10010200, Jena Bioscience) cells were incubated with 0.2% Triton X-100 solution in PBS 1X for 15 min at room temperature. After three 0.05% TBST washes for 5 min, cells were blocked with 3% FBS in PBS 1X for 30 min and incubated with primary antibody as described above. Secondary antibodies incubation was performed at room temperature for 1 hour (1:200; Alexa Fluor® 488-conjugated AffiniPure Goat anti-rabbit IgG, cat. no. 111-545-003, Jackson Immunoresearch Laboratories Inc., USA and 1:2000; Alexa Fluor™ 568 goat anti-mouse IgG H + L, cat. no. A11031, Invitrogen, Life Technologies Corporation) in buffers used for blocking. HOECHST 33342 (cat. no. H-3570; Molecular Probes, USA) stained slides were washed in PBS and mounted with Vectashield mounting medium (H-1200, Vector Laboratories). Confocal analysis was performed using a Nikon A1 confocal laser scanning microscope, equipped with a SR Apo TIRF 100×/1.49 NA objective lens and with 405, 488, and 561 nm laser lines. Z-stacks were collected at an optical resolution of 80 nm/pixel, stored at 12-bit with 4096 different grey levels pinhole diameter set to 1 Airy unit, and z-step size to 200 nm. Confocal images were processed using the Richardson-Lucy deconvolution algorithm by using NIS-Elements Advanced Research software (Nikon, Tokyo, Japan). A large area of the samples was observed, and 5 representative cells for each condition were analyzed and processed as described thereafter. Colocalization analysis was evaluated by comparing the equivalent pixel positions of blue and red signals of fluorophores in each of the acquired images (optical sections). A two-dimensional scatter plot diagram of the individual pixels from the paired images was generated and a threshold level of signal to be included in the analysis was selected. Pixels with intensity values greater than 50% grey levels (on a scale from 0 to 4096) were selected for both signals, and the colocalization binary maps that indicate regions containing highly colocalized signals were imaged and merged (in white) to the blue and red signals[51, 52]. The levels of pH2A.X and cytoplasmic nucleic acids immunofluorescence intensity were evaluated semi-quantitatively by measuring the mean fluorescence intensity (MFI) in representative regions of interest within the nucleus or cytoplasm. The measurements use single optical sections through the middle of the nucleus. The analysis of the images was carried out using NIS-Elements HC software version 5.20 (Nikon, Tokyo, Japan). The statistical significance of differences between experimental points was determined using Student’s t test.

### Preparation of RNA-seq libraries

RNA for the transcriptomic assay was extracted with RNeasy Mini Kit by Qiagen (cat. no. 74104) following the manufacturer’s standard protocol. The quality of extracted material was assessed with NanoDrop spectrophotometer (Thermo Fisher Scientific, US). All RNA samples were purified using RNAClean XP beads by Beckman Coulter (cat. no. A63987) according to the manufacturer’s instructions. The efficiency of the purification and final quality control was performed with i) NanoDrop and ii) Qubit fluorometer (Thermo Fisher Scientific, US) using Qubit RNA Broad Range Assay Kit (cat. no. Q10210, Invitrogen). RNA was stored at −80 °C until preparation of the RNAseq libraries. The abundance of ribosomal RNA was removed from each sample using NEBNext rRNA Depletion Kit v2 (Human/Mouse/Rat) by New England BioLabs Inc. (cat. no. E7405) following the standard H-based workflow indicated in the product manual and using as input 200 ng of sample RNA. Further, RNA-seq libraries were prepared with NEBNext Ultra II Directional RNA Library Prep Kit for Illumina (cat. no. E7760S, New England BioLabs Inc.) according to the manufacturer’s instructions. Each library was identified with a unique index selected from a set of NEBNext Multiplex Oligos for Illumina (96 Unique Dual Index Primer Pairs, cat. no. E6440S, New England BioLabs Inc.). Quality and size of libraries (~300 bp) were confirmed using) Qubit fluorometer (Thermo Fisher Scientific, US) with Qubit DNA Broad Range Assay Kit (cat. no. Q32853, Invitrogen) and ii) Tape Station with D100 ScreenTape device (cat. no. 5067-5582, Agilent Technologies Inc.). Libraries were pooled and stored at −20 °C until sequencing. The entire pool was sequenced on NovaSeq 6000 Illumina system.

### Transcriptomic analysis

Raw *fastq* files were processed on Illumina DRAGEN Bio-IT Platform 3.7 following a standardized RNA-seq analysis pipeline. Gene expression quantification was performed estimating the expression of each transcript and gene in an RNA-seq dataset by translating the genomic mapping of each read to the corresponding transcript mappings and then using Expectation-Maximization (EM) algorithm inferring the transcript expression values that best match all the observed reads. Differential expression analysis was performed in R environment (version 4.2.2) using as an input count table corresponding to genes. RNA-seq dataset was transformed to counts per million (CPM) and filtered removing lowly expressed events – only genes with more than 10 reads in at least two samples were kept. No outliers were detected thus all the samples presented average read count within the range <GroupMean-3*SD; GroupMean+3*SD>.

### Transmission electron microscopy

For post-embedding immunoelectron microscopy, cells were fixed with 1% glutaraldehyde in phosphate buffer and embedded in Epon812 resin. Ultrathin sections were incubated for 25 min in PBS (pH 7.2) containing normal goat serum (NGS) diluted 1:30 and then with mouse monoclonal anti-dsDNA antibodies diluted 1:10 in PBS and revealed with goat anti-mouse 10 nm colloidal gold conjugated antibody (Sigma, G7652) diluted 1:100 in PBS for 60 min at room temperature. This labeling was systematically applied to both faces of the ultrathin sections. Samples were then rinsed with PBS followed by distilled water. Sections were stained with lead citrate and observed with a Jeol JEM-1011 transmission electron microscope operated at 100 kV. Several kinds of control experiments were carried out. When the primary or secondary antibody or both were omitted, the ultrathin sections were devoid of label. When the grids were incubated with antibody-free particles, no labeling occurred.

### RNase III and RNase T1 enzymatic treatment

Formaldehyde-fixed samples were washed once with PBS, and then incubated in permeabilization buffer (PBS with 0.1% Triton X-100) for 10 min. Samples were then incubated in a staining buffer (TBST with 0.1% BSA) for 10 min with rocking. Enzymatic treatments were done in staining buffer supplemented with 3 mM magnesium chloride with 1:200 dilutions of RNase T1 (cat. no. EN0541, Thermo Fisher), RNase III (ShortCut RNase III, cat. no. M0245S, New England Biolabs Inc.) and incubated with rocking for 1 h. Samples were subsequently washed with staining buffer for 10 min with rocking and incubated overnight with primary antibody.

### Extracellular vesicle isolation and characterisation

EVs were isolated as previously described [27]. EV surface antigens were investigated with the MACSPlex Exosome kit (Miltenyi Biotec GmbH, Gladbach, Germany). Briefly, after isolation, EVs were diluted in MACSPlex buffer and stained according to manufacturer instructions. The samples were analyzed with a FACSCanto flow cytometer (Beckton Dickinson). At least 10,000 events per sample were recorded. Data were analyzed with FACSDiva software.

### Dot blot

Total RNA was resuspended to a final volume of 50 μl in nuclease-free water and spotted directly onto nylon Hybond N+ membrane (GE Healthcare) using a Bio-Dot Apparatus (Bio-Rad, Hercules, CA). The membrane was UV-cross-linked and blocked with PBS with 5% BSA and 0.1% Tween-20 before incubation with primary and secondary antibodies. A 5 μg aliquot of m6A antibody (SYSY Synaptic Systems) for N6-methyladenosine-modified RNA was used for the primary, and a 20,000x dilution of goat anti-mouse HRP (Bio-Rad) was used as the secondary antibody, respectively. The HRP signal was visualized using Clarity Western ECL Substrate (BioRad) and the ChemiDoc MP Imaging System (BioRad).

### Statistical analysis

Data are presented as the mean ± SD from three replicates. All data were processed by GraphPad Prism 9.4 software. Unpaired or paired Student’s *t* test and one-way analysis of variance were used to determine the statistical differences among different treatments. A *P*-value < 0.05 was considered statistically significant.

For transcriptome data analysis, the expression counts were normalized accounting for library sizes (*edgeR* Bioconductor R package) and log-CPM mean-variance fitted values were used in linear modeling (*limma* Bioconductor R package). Moderated t-statistics of differential expression were calculated using the empirical Bayes approach and obtained *p*-values were corrected for multiple testing with the Benjamini–Hochberg procedure. The genes that reached a statistical significance level of 0.01 with adjusted p-value and exceeded the threshold of 1.5 with *log2* fold change (logFC) of absolute expression, were considered as differentially expressed (DE). Four contrasts were considered in the RNA-seq analysis design:

- Senescent *vs* young cells cultivated in normal glucose levels—allowing to identify changes in gene expression related to Senescence in normal glucose condition,
- Senescent *vs* young cells cultivated in high glucose levels—determining genes related to Senescence in high glucose condition,
- Senescent cells cultivated in normal *vs* high glucose medium—indicating changes related to high glucose condition in Senescent cells and
- young cells cultivated in normal *vs* high glucose medium—identifying transcripts related to high glucose condition in young cells.

Additionally, to confirm the coherence of results that emerged from DE analysis, genes were cross-checked in external on-line resources dedicated to senescence. We have consulted Human Ageing Genomic Resources, *HAGR CellAge* database (available at https://www.genomics.Senescence.info/) consisting of manually-curated collection of human genes associated with cellular Senescence in vitro plus a gene expression signature of replicative senescence derived from a meta-analysis of human microarray data [53, 54], and *SeneQuest* (available at http://Senequest.net) which is an exhaustive resource tool gathering and summarizing the information on gene-to-Senescence associations emerging from up-to-date publications [17]. Furthermore, to provide a complete interpretation of RNA-seq results, pathway enrichment analysis was performed employing *Reactome* database (available at https://reactome.org/) - an open access, manually curated and peer-reviewed pathway repository [55].

### Supplementary information

Supplementary Table 1

Supplementary Table 2

Supplementary Figure 1

Supplementary Figure 2

Original Data File

## Data Availability

The RNA-seq datasets are available in the ArrayExpress repository, under the accession number E-MTAB-13959 (https://www.ebi.ac.uk/arrayexpress/experiments/E-MTAB-13959/). The other datasets generated during and/or analysed during the current study are available from the corresponding author on reasonable request.
