# Supplementary material for: Replicative senescence and high glucose induce the accrual of self-derived cytosolic nucleic acids in human endothelial cells
Source: Cell Death Discov. 2024 Apr 20;10:184. doi: 10.1038/s41420-024-01954-z (PMC11032409; doi:10.1038/s41420-024-01954-z)

Supplementary Figure 1. MDS plot showing the relationships between expression profiles of common senescence-related genes (set A) in all 12 samples used in RNA-seq experiment. Each label represents a single sample. In green: young cells cultivated in normal glucose; in orange: young cells cultivated in high glucose medium; in blue: senescent cells cultivated in normal glucose medium; in purple: senescent cells cultivated in high glucose medium.

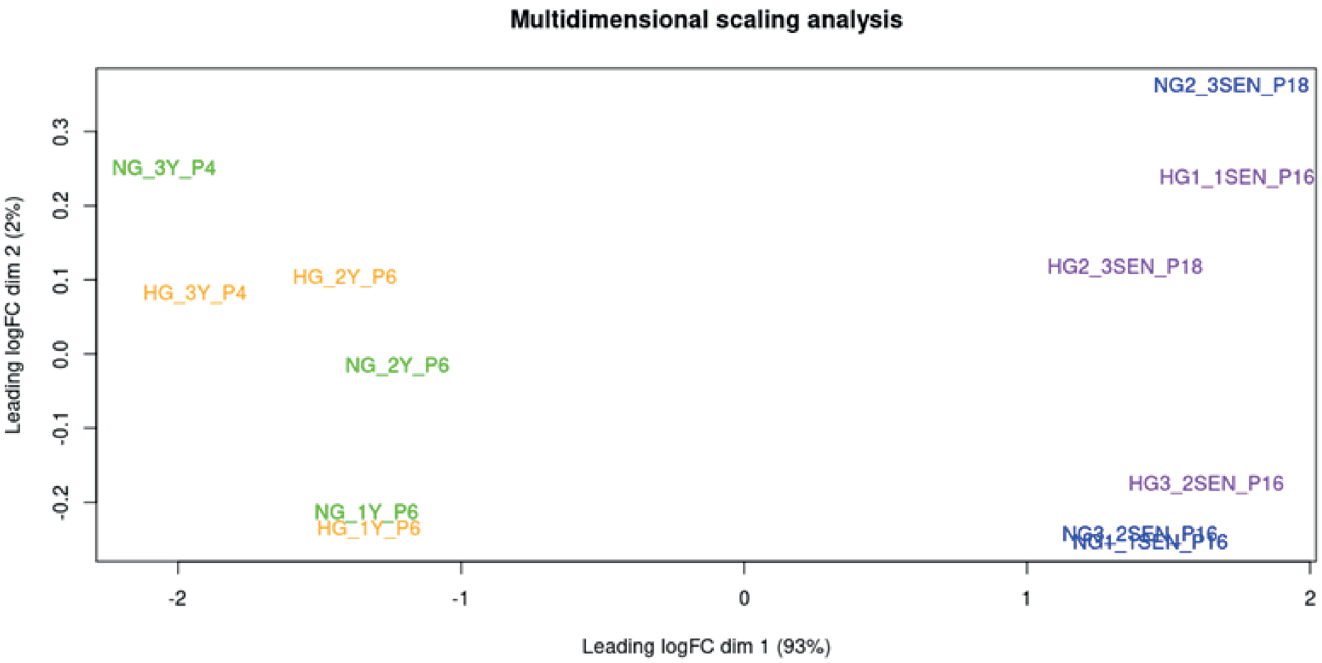

Supplement: Supplementary file 3 — Supplementary Figure 1 [file 41420_2024_1954_MOESM3_ESM.pdf]
