# Supplementary material for: Replicative senescence and high glucose induce the accrual of self-derived cytosolic nucleic acids in human endothelial cells
Source: Cell Death Discov. 2024 Apr 20;10:184. doi: 10.1038/s41420-024-01954-z (PMC11032409; doi:10.1038/s41420-024-01954-z)

Supplementary Figure 2. (A) Quantification of 3 different EV markers obtained by MACSPlex Assays in HUVEC cells. (B) Average size distribution curve of sEVs released from Ctr and Ctr-HG (upper panel) and Sen and Sen-HG (lower panel). EVs size was determined by Nanoparticle Tracking Assay (NTA). Three 60 s videos were recorded for each sample, and NTA analysis settings kept constant between samples. The average concentration of vesicles was plotted against their size, with the black lines and the colour areas representing the fitting curve and the error bar, respectively.

A

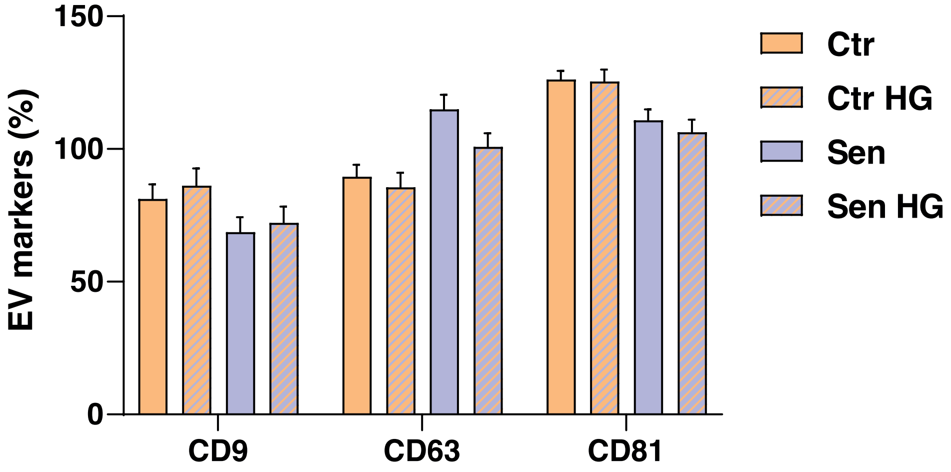

B

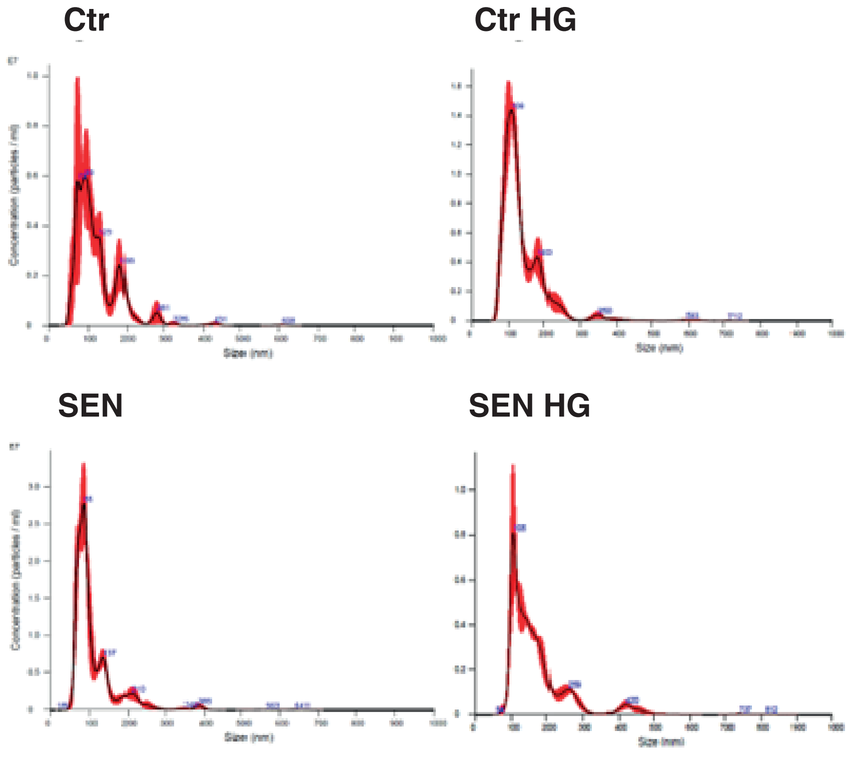

Supplement: Supplementary file 4 — Supplementary Figure 2 [file 41420_2024_1954_MOESM4_ESM.pdf]
