# Supplementary material for: Replicative senescence and high glucose induce the accrual of self-derived cytosolic nucleic acids in human endothelial cells
Source: Cell Death Discov. 2024 Apr 20;10:184. doi: 10.1038/s41420-024-01954-z (PMC11032409; doi:10.1038/s41420-024-01954-z)

Figure 1C (original blot)

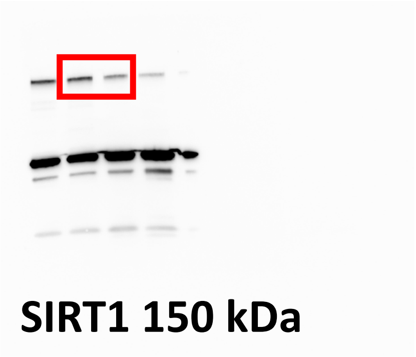

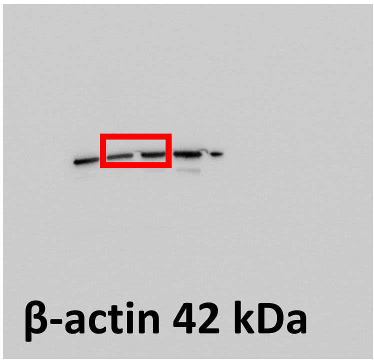

Figure 1D

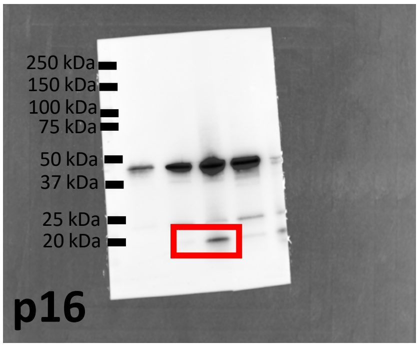

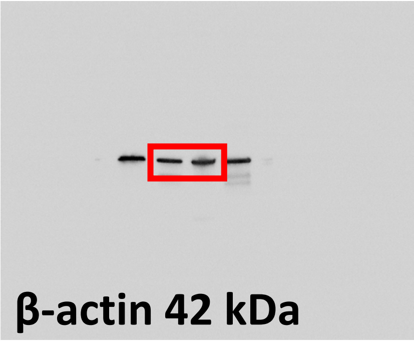

Figure 2E (original blot)

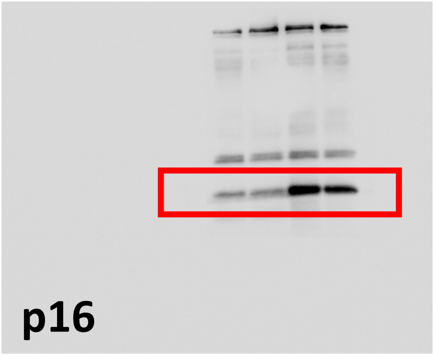

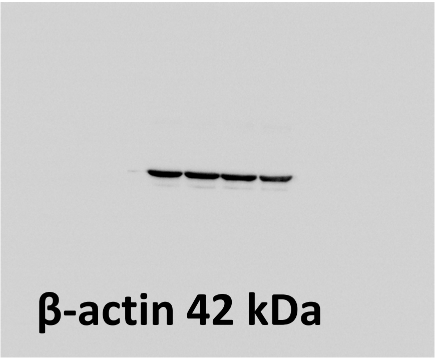

Figure 4D (original blot)

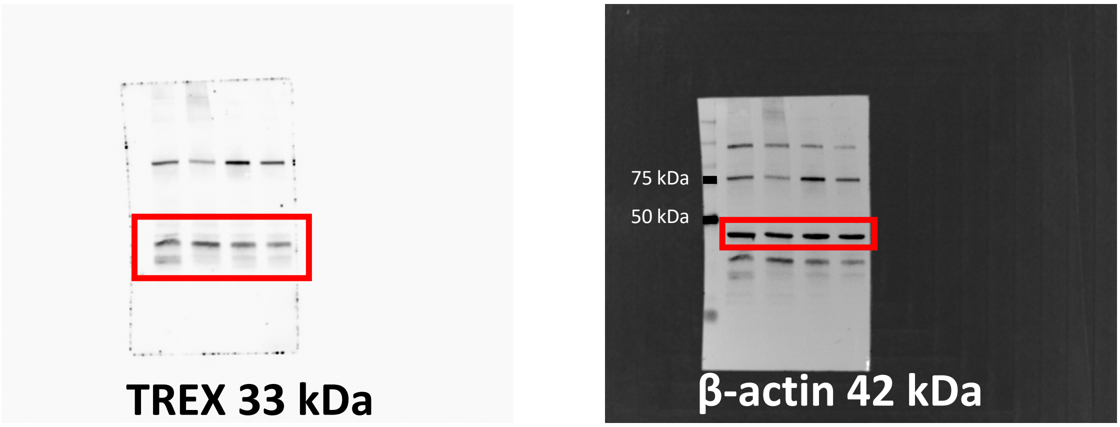

Figure 5A (original blot)

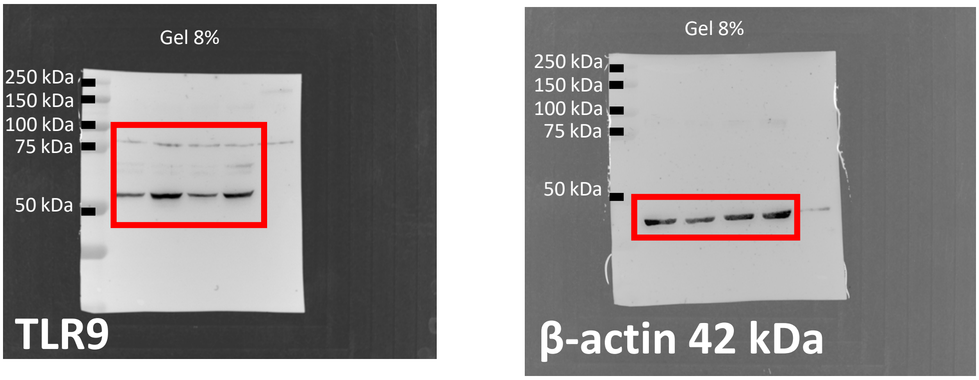

Figure 5B (original blot)

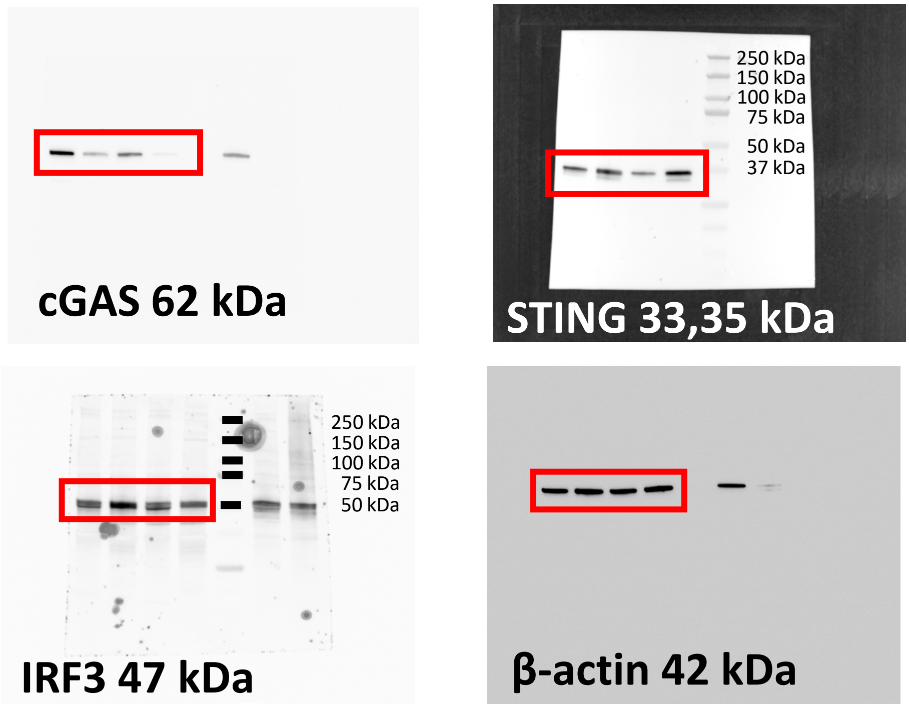

Figure 5C (original blot)

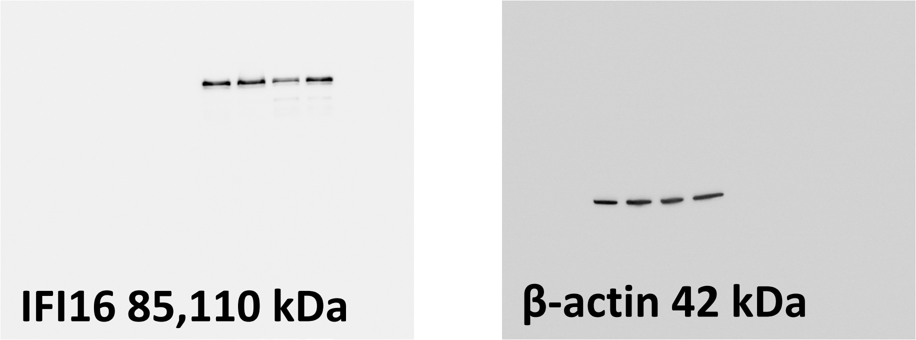

Figure 5E (original blot)

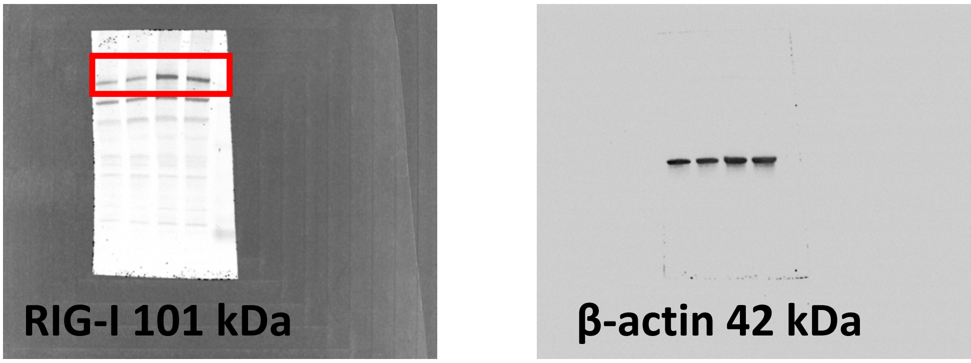

Supplement: Supplementary file 5 — Original Data File [file 41420_2024_1954_MOESM5_ESM.pdf]
